# Supplementary material for: Evolution of the Family Equidae, Subfamily Equinae, in North, Central and South America, Eurasia and Africa during the Plio-Pleistocene
Source: Biology (Basel). 2022 Aug 24;11(9):1258. doi: 10.3390/biology11091258 (PMC9495906; doi:10.3390/biology11091258)
Supplement: Supplementary file 1 [file biology-11-01258-s001.zip › Equinae ms_Supplementary Text.pdf]

biology

MDPI

Supplementary

Evolution of the Family Equidae, Subfamily Equinae, in North, Central and South America, Eurasia and Africa during the Plio-Pleistocene

Omar Cirilli [1,2], Helena Machado [3**], Joaquin Arroyo-Cabrales [4], Christina I. Barrón-Ortiz [5], Edward Davis [3,6], Christopher N. Jass [5], Advait M. Jukar [7,8,9], Zoe Landry [10], Alejandro H. Marín-Leyva [11], Luca Pandolfi [12], Diana Pushkina [13], Lorenzo Rook [2], Juha Saarinen [13], Eric Scott [14,15], Gina Semprebon [16], Flavia Strani [17,18], Natalia A. Villavicencio [19,20,21], Ferhat Kaya [13,22*] and Raymond L. Bernor [1,23*]**

1 Laboratory of Evolutionary Biology, College of Medicine, Department of Anatomy, Howard University, 20059, Washington DC, USA
2 Earth Science Department, Paleo[Fab]Lab, University of Florence, via La Pira 4, 50121, Firenze, Italy
3 University of Oregon, Earth Sciences Department, 100 Cascade Hall, Eugene, 97403-1272, OR, United States
4 Instituto Nacional de Antropología e Historia Laboratorio de Arqueozoología "M. en C. Ticul Álvarez Solórzano", Subdirección de Laboratorios y Apoyo Académico, Ciudad de México, Mexico
5 Quaternary Palaeontology Program, Royal Alberta Museum, 9810 103a Ave NW, Edmonton, Alberta T5J 0G2, Canada
6 Clark Honors College, University of Oregon, Eugene, OR, United States
7 Department of Geosciences, University of Arizona, 1040 E 4th St. Tucson AZ 85721
8 Department of Paleobiology, National Museum of Natural History, Smithsonian Institution, 10th St and Constitution Ave NW, Washington DC 20013
9 Division of Vertebrate Paleontology, Yale Peabody Museum of Natural History, 170 Whitney Ave, New Haven CT 06520
10 Department of Earth Sciences, University of Ottawa, 25 Templeton Street, Ottawa, Ontario K1N 6N5, Canada
11 Laboratorio de Paleontología, Facultad de Biología, Universidad Michoacana de San Nicolás de Hidalgo. Edif. R 2°. Piso. Ciudad Universitaria, Morelia, Mexico
12 Department of Science, University of Basilicata, Via dell'Ateneo Lucano, 10 - 85100, Potenza, Italy
13 Department of Geosciences and Geography, University of Helsinki, Helsinki, Finland
14 Cogstone Resource Management, Inc., 1518 W. Taft Avenue, Orange, 92865, CA, United States
15 Department of Biology, California State University, 5500 University Parkway, San Bernardino, 92407, CA, United States
16 Bay Path University, 588 Longmeadow Street, Longmeadow, 01106, MA, United States
17 Dipartimento di Scienze della Terra, Laboratorio PaleoFactory, Sapienza Università di Roma, Piazzale Aldo Moro 5, 00185 Rome, Italy
18 Departamento de Ciencias de La Tierra, and Instituto Universitario de Investigación en Ciencias Ambientales de Aragón (IUCA), Universidad de Zaragoza, 50009, Zaragoza, Spain;
19 Corporación Laguna de Taguatagua, Av. Libertador Bernardo O'Higgins 351, Santiago, Chile;
20 Instituto de Ciencias Sociales, Universidad de O'Higgins, Av. Libertador Bernardo O'Higgins 611, Rancagua, Chile
21 Biomolecular Laboratory, Institut Català de Paleoecologia Humana i Evolucio Social, Tarragona, 43007, Spain
22 Department of Archaeology, University of Oulu, Oulu, Finland
23 Human Origins Program, Department of Anthropology, National Museum of Natural History, Smithsonian Institution, 20013, Washington, DC, USA
* Correspondence: FK kaya.ferhat@oulu.fi ; RLB rbernor@howard.edu ; rlbernor@gmail.com; BernorR@SI.edu
** OC and HM are co-first authors of the present manuscript. .
*** RLB is the senior author of this manuscript

Simple Summary: The family Equidae enjoys an iconic evolutionary record, and especially the genus *Equus* which is actively investigated by both paleontologists and molecular biologists. Nevertheless, a comprehensive evolutionary framework for *Equus* across its geographic range including North, Central and South America, Eurasia and Africa is long overdue. Herein, we provide an updated taxonomic framework so as to develop its biochronologic and biogeographic frameworks that lead to well resolved paleoecologic, paleoclimatic and phylogenetic interpretations. We present *Equus'* evolutionary framework in direct comparison to more archaic lineages of Equidae that coexisted, but progressively declined over time, alongside evolving *Equus* species. We show the varying correlations between body size, and we use paleoclimatic map reconstructions to show the environmental changes accompanying taxonomic distribution across *Equus* geographic and chronologic ranges. We present the two most recent phylogenetic hypotheses on the evolution of the genus *Equus* using osteological characters and address parallel molecular studies.

Abstract: Studies of horse evolution arose during the middle of the 19th Century, and several hypotheses have been proposed for their taxonomy, paleobiogeography, paleoecology and evolution. The present contribution represents a collaboration of 19 multinational experts with the goal of providing an updated summary of Pliocene and Pleistocene North, Central and South American, Eurasian and African horses. At the present time, we recognize 114 valid species across these continents, plus 4 North African species in need of further investigation. Our biochronology and biogeography sections integrate Equinae taxonomic records with their chronologic and geographic ranges recognizing regional biochronologic frameworks. The paleoecology section provides insights on paleobotany and diet utilizing both the mesowear and light-microscopic methods, along with calculation of body masses. We provide a temporal sequence of maps that render paleoclimatic conditions across these continents integrated with Equinae occurrences. These records reveal a succession of extinctions of primitive lineages and the rise and diversification of more modern taxa. Two recent morphological based cladistic analyses are presented here as competing hypotheses, with reference to molecular-based phylogenies. Our contribution represents a state-of-the art understanding of Plio-Pleistocene *Equus* evolution, their biochronologic and biogeographic background, paleoecological and paleoclimatic contexts.

Keywords: Equidae; Equinae; Hipparionini; Protohippini; Equini; Paleoecology; Paleoclimatology; Biochronology; Phylogeny; Evolution.

3. *Systematics of the Equinae since 5.3 Ma in North and South America*

3.1. Taxonomy of North and Central American **Equus** *sensu lato: Introduction and Objectives*

The taxonomy of North and Central American *Equus* in the broad sense (sensu lato) has a long and complex history. There is a lack of taxonomic consensus at virtually every level, from species to genera (for example see Gidley, 1901; Schultz, 1936; Savage, 1951; Quinn, 1957; Skinner, 1972; Dalquest, 1978; Bennett, 1980; Winans, 1985, 1989; Azzaroli and Voorhies, 1993; Azzaroli, 1998; Pichardo, 2000; Scott, 2004; Eisenmann et al., 2008; Barrón-Ortiz et al., 2017; Heintzman et al., 2017).

Given the large number of studies discussing fossil specimens of North and Central American *Equus* sensu lato and the wide range of opinions regarding the taxonomy of this group of equids, we saw the need to summarize the taxonomic conclusions of most of the relevant studies that have been published from 1901 to 2021. We identified 68 relevant studies (Table S6A). Although our review is comprehensive, it is not exhaustive and we acknowledge that we may have inadvertently missed relevant studies. We summarized the taxonomic conclusions of the 68 studies in Table S6A. Using this information, we created taxa-concept-maps with two objectives in mind: (1) to visually summarize the taxonomic conclusions at the species and subspecies levels of the 68 studies we surveyed and (2) to use the information presented in the taxa-concept-maps to identify potentially valid species. A description of the methodology we followed is presented below.

Using the information presented in the taxa-concept-maps, we identified 16 potentially valid species of North and Central American *Equus* sensu lato. We view the potentially valid species that we recognized as taxonomic hypotheses that can be tested with further morphological and molecular studies. We hope that the information we present will serve as a useful reference for future studies on the evolution and taxonomy of *Equus* sensu lato.

3.2. ***Table S6A: Summary of taxonomic conclusions***

We conducted a literature survey of studies discussing fossil specimens of North and Central American *Equus* sensu lato. Our literature survey focused on studies spanning a timeframe from 1901 to 2021 (n = 68 studies; Table S6A). We summarized the taxonomic conclusions of these 68 studies in Table S6A . Each row in Table S6 corresponds to a named, referred (i.e., cf.), or, in some cases, unnamed or indeterminate taxon of *Equus*. The first column indicates the genus and, if applicable, the subgenus of a taxon as it was originally named. The second column shows the species and, if applicable, subspecies name of a taxon as it was originally named. The third column shows the author(s) and year that the taxon was named. The remaining columns show the taxonomic conclusions of each study that we surveyed.

3.3. ***Taxa-concept-maps for species and subspecies***

We summarized the taxonomic conclusions at the species and, if applicable, subspecies level of the studies we surveyed by creating taxa-concept-maps (Figures S1.1 – S1.3). We did not consider referred and unnamed/indeterminate species in the elaboration of the taxa-concept-maps, except for *E. caballus/ferus* and *E. kiang* where we considered referred specimens.

In the taxa-concept-maps, species and subspecies names are shown with numbers at the bottom of each name to indicate the number of times that the name was considered valid at the species level (and if applicable at the subspecies level), and the number of times that the name was considered a *nomen dubium/nomen nudum* (at the species level and, if applicable, at the subspecies level). We present this information following this

format: name considered valid at the species level - name considered valid at the subspecies level (if the name was used at the subspecies level) / name considered *nomen dubium/nomen nudum* at the species level - name considered *nomen dubium/nomen nudum* at the subspecies level (if the name was used at the subspecies level). For example, *scotti* 18-1/0-0. This indicates that *scotti* has been considered a valid species name 18 times, a valid subspecies name 1 time, a *nomen dubium/nomen nudum* at the species level 0 times, and a *nomen dubium/nomen nudum* at the subspecies level 0 times.

In some cases, the validity of a name was questionable. We indicated the number of times a name was considered questionable with a question mark. For example: *parastylidens* 6,2?/1. This indicates that *parastylidens* has been considered a valid name at the species level 6 times, a questionably valid name at the species level 2 times and a *nomen dubium/nomen nudum* at the species level 1 time. Note that in this case this name has not been used at the subspecies level.

For taxa that have both specific and subspecific names (for example, *E. niobrarensis alaskae*), we only evaluated whether the subspecific name has been considered valid at the species level and the subspecies level, and whether it has been considered a *nomen dubium/nomen nudum* at the species level and subspecies level. We took this approach because most studies elevate the subspecies name to species rank. For example, *niobrarensis alaskae* 3-1/0-0. This indicates that *alaskae* has been considered a valid species name 3 times, a valid subspecies name 1 time, a *nomen dubium/nomen nudum* at the species level 0 times, and a *nomen dubium/nomen nudum* at the subspecies level 0 times.

In the taxa-concept-maps, we also indicate taxonomic synonymies or reidentification of specimen samples with arrows. Note that this is only done at the species level and that we did not separate synonyms from reidentification of specimen samples because there were several studies in which it was not clear whether the author(s) were identifying particular specimen samples under a different species name or whether they were implying that the names should be synonymized. The number beside a particular arrow indicates the number of times that a specific name has been synonymized with another name and/or the number of times a sample identified under a particular species name was reidentified as another name. For example, there is one arrow that goes from *francisci* to *tau* with the numbers 4/1? next to it. This indicates that *francisci* has been synonymized with and/or samples assigned to *francisci* have been reidentified as *tau* 4 times and in 1 instance the synonymy or reidentification is probable or questionable.

Collectively, the arrows and the numbers beside the arrows show the degree of association between different names. The degree of association between two names is represented by three types of arrows to visually indicate whether the association is weak (mentioned in 1 or 2 studies), moderate (mentioned in 3 or 4 studies), or strong (mentioned in 5 or more studies) (Figures S1.1 – S1.3).

The taxa-concept-maps also show the relative completeness of type specimens for each name and whether a type was not named or if it has been lost.

Lastly, we indicate names that we think should not be considered in the interpretation of North and Central American fossil horse remains. Currently, there is strong morphological and molecular evidence to suggest that the type specimens of some names are historic in age (e.g., *Equus laurentius*), some species did not occur in North and Central America (e.g., *Equus kiang*), and some species make their appearance only during historic times (e.g., *Equus asinus*) as a result of human introduction (e.g., Weinstock et al., 2005; Scott, 2010; Eisenmann et al., 2008; Heintzman et al., 2017).

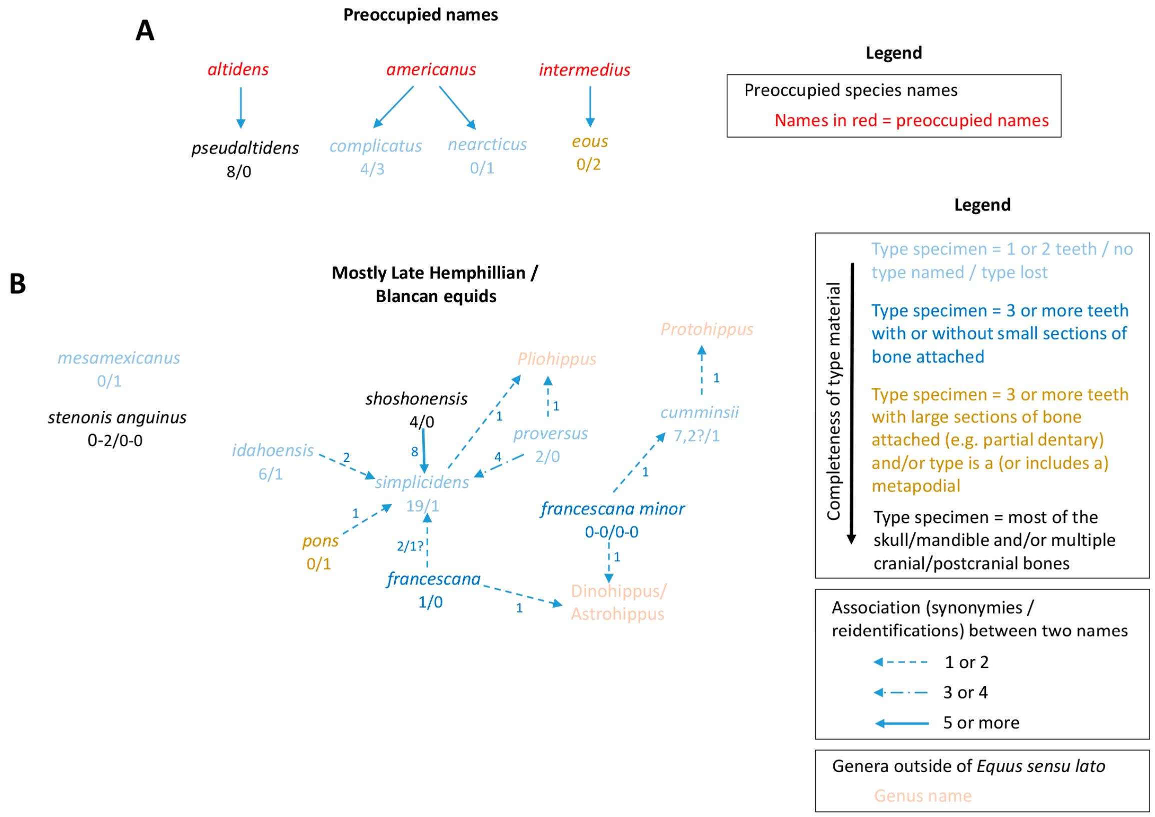

Figure S1.1 (A) Preoccupied names of *Equus* sensu lato and substitute names. (B) Taxa-concept-map of *Equus* sensu lato showing mostly Late Hemphillian / Blancan equids, based on the studies we evaluated. Refer to Section 3.3 for explanation.

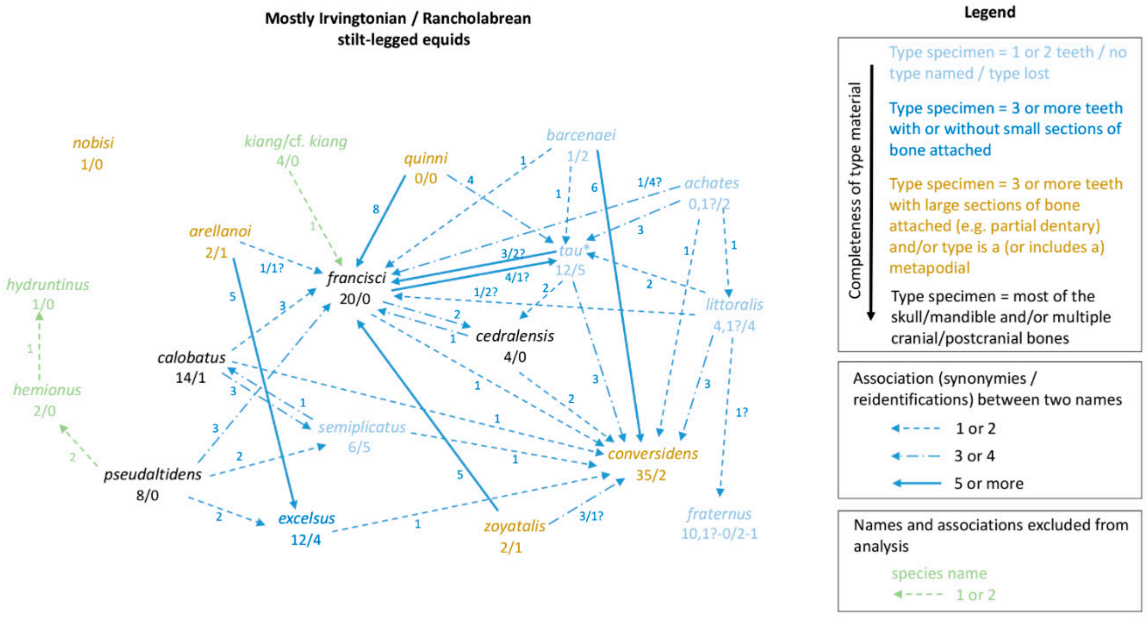

Figure S1.2 Taxa-concept-map of *Equus* sensu lato showing mostly Irvingtonian / Rancholabrean stilt-legged equids, based on the studies we evaluated. Refer to Section 3.3 for explanation. *The type specimen of *E. tau* is lost. Eisenmann et al. (2008) proposed as neotype of *E. tau* a partial skull from Aguascalientes, Mexico, currently housed at the

University of Texas at Austin, but this proposal is rejected by some authors (Heintzman et al., 2017; Priego-Vargas et al., 2017).

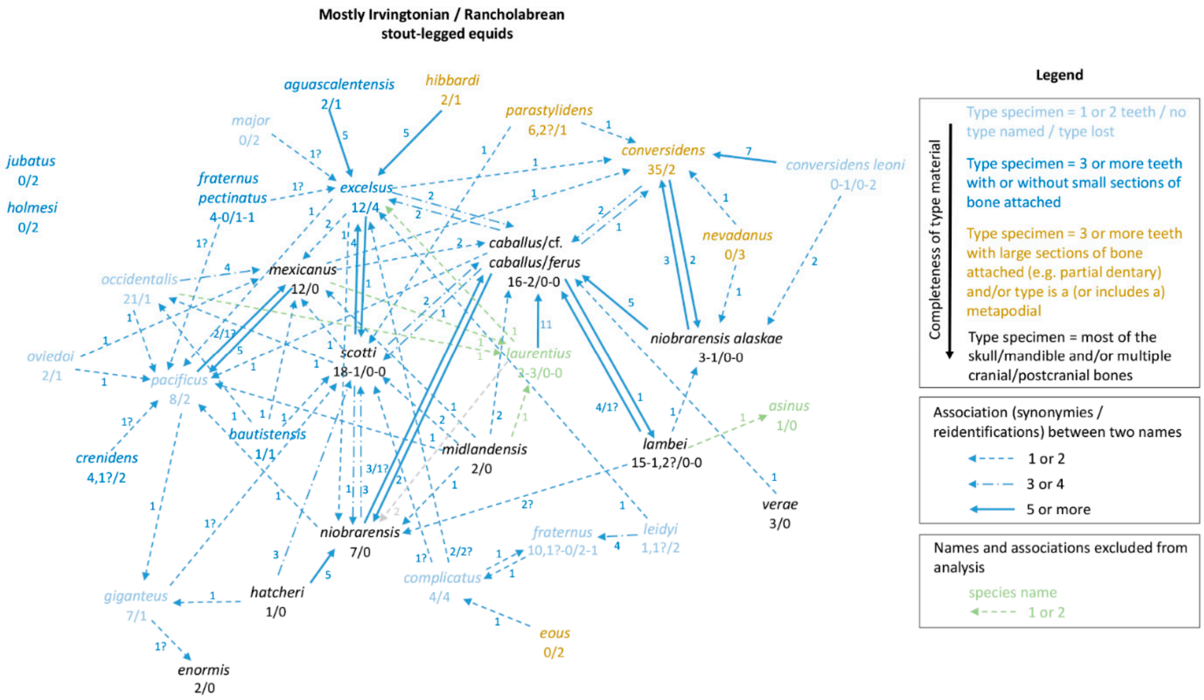

Figure S1.3 Taxa-concept-map of *Equus* sensu lato showing mostly Irvingtonian / Rancholabrean stout-legged equids, based on the studies we evaluated. Refer to Section 3.3 for explanation.

3.4. *Identifying potentially valid species*

The taxa-concept-maps we created revealed the presence of three distinct clusters of names. The first cluster consists of mostly Late Hemphillian - Blancan species (Figure S1.1B). The second cluster consists of mostly Irvingtonian - Rancholabrean stilt-legged species (Figure S1.2). The third cluster consists of mostly Irvingtonian - Rancholabrean stout-legged species (Figure S1.3).

To identify potentially valid horse species within each cluster, we evaluated the number of times that a particular name was considered valid relative to the number of times that the name was considered a *nomen dubium*/*nomen nudum* and the number of times the name was synonymized/reidentified with another name. In other words, we performed the following calculation: number of times a particular name was considered valid - (number of times the name was considered a *nomen dubium*/*nomen nudum* + number of times the name was synonymized/reidentified with another name). For example, *lambei* was considered a valid name at the species level 14 times, it was considered a *nomen dubium*/*nomen nudum* at the species level 0 times, and it was synonymized/reidentified with another name 5 times (not considering questionable synonymies/reidentifications): 14 - (0 + 5) = 9. We performed these calculations for every name. The results ranged from 29 for *conversidens* to -12 for *quinni* (Table S6B). Only 23 out 55 names analyzed had positive "validity" scores (Table S6B).

Four names had scores that were >10: *conversidens*, *simplicidens*, *occidentalis*, and *francisci*. These results suggest that most studies that we surveyed considered these four species as valid. The last name, *francisci*, is based on a relatively complete type specimen (Hay, 1915). Although *simplicidens* and *occidentalis* are each based on an isolated cheek tooth (Gidley, 1901) and *conversidens* is based on a palate with associated left and right cheek teeth (Owen, 1869; Hibbard, 1955), large samples of specimens have been referred

to each of these taxa (e.g., Hagerman, Rancho La Brea, and San Josecito Cave, respectively) (Azzaroli and Voorhies, 1993; Brown et al. 2015; Barrón-Ortiz et al., 2017). We consider these four species as potentially valid.

Seven names had scores that ranged from 10 – 6: *lambei*, *scotti*, *caballus/ferus*, *fraternus*, *calobatus*, *cumminsii*, and *giganteus*. These results suggest that a large portion of studies we surveyed considered these species as valid. The first two names, *lambei* and *scotti*, are based on relatively complete type specimens (Gidley, 1900; Hay, 1917). The taxa-concept-map points to a strong association between *caballus/ferus* and large extinct caballines (e.g., *niobrarensis* and *scotti*) on one side and small caballines (e.g., *lambei* and *niobrarensis alaskae*) on the other (Figure S1.3). The strong association between these names may indicate that large and small North American extinct caballines are synonymous with *caballus/ferus* or that they are distinct but closely related species. Morphologically, large and small North American caballines differ from each other in some traits (e.g., Azzaroli, 1998; Barrón-Ortiz et al., 2017). Genetically, there is some evidence to suggest that they are closely related taxa, if not conspecific (e.g., Orlando et al. 2013; Barrón-Ortiz et al., 2017; Vershinina et al. 2021). Further studies are needed to clarify the evolutionary relationship between large and small North American caballines and their relationship with *E. ferus*. For the purposes of this study, we retain *scotti* and *lambei* as distinct species, given that the taxa-concept-map does not reveal a direct association between these two names and each one has a high "validity" score.

Although *fraternus* is based on isolated cheek teeth and perhaps other fragmentary remains (Gidley, 1901; Winans, 1985), several complete skulls, mandibles, and other bones have been referred to this species (e.g., Azzaroli, 1995, 1998). Therefore, we consider this species as potentially valid. Likewise, we consider *calobatus* and *cumminsii* as potentially valid species. The type of *calobatus* consists of an associated postcranial skeleton (Winans, 1985) and several specimens from various localities across North America have been referred to this species (Kurtén and Anderson, 1980). Although the type specimens of *cumminsii* and *simplicidens* are both isolated upper cheek teeth (Gidley, 1901), the taxa-concept-map shows that there is no direct association between these two names and that several authors consider them distinct species (Figure S1.1B). One of the primary distinctions between *cumminsii* and *simplicidens* is in tooth dimensions (Gidley, 1901; Scott, 2004), but other potentially relevant differences lie in the development of the hypoconal groove and the shape and development of the mesostyle. Lower cheek teeth referred to *cumminsii* and *simplicidens* differ in the development of the ectoflexid of the lower molars (Dalquest, 1978; Scott, 2004).

Regarding *giganteus*, this name was based on an isolated tooth, whose primary diagnostic trait was its unusually large size (Gidley, 1901). The taxonomic utility of this trait has been questioned by some authors (e.g., Winans, 1985; Dalquest and Schultz, 1992). Moreover, there are no large samples of specimens that have been referred to this species and most records consist of isolated teeth, particularly upper premolars (Dalquest and Schultz, 1992). Therefore, we consider *giganteus* a doubtful name.

Twelve names had scores that ranged from 4 – 1: *mexicanus*, *idahoensis*, *pacificus*, *parastylidens*, *pectinatus*, *enormis*, *stenonis anguinus*, *verae*, *crenidens*, *cedralensis*, *pseudaltidens*, and *nobisi*. Three of these names (*parastylidens*, *pectinatus*, and *crenidens*) are considered doubtful names because the taxonomic utility of the traits on which they were based has been questioned (e.g., Winans, 1985) and there are no large samples of specimens that have been referred to either of those species that could supplement their diagnosis.

Out of the remaining names, the validity of *pacificus* and *nobisi* is also questionable. The type specimen of *pacificus* is an isolated cheek tooth that was described as not strikingly different from corresponding teeth of living horses except for its large size (Winans, 1985). Some samples have been referred to this species, but they consist mostly of isolated or associated teeth (e.g., Gidley, 1901; Lundelius, 1972b). Tooth size, by itself,

is an unreliable taxonomic character (Winans, 1985); thus, for the present, we consider *pacificus* a doubtful name. The taxa-concept-map indicates a strong association between *pacificus* and *mexicanus* (Figure S1.3), suggesting that many authors of the studies we surveyed consider them synonymous. A comprehensive study of both holotypes and referred specimens is necessary to clarify this issue.

Equus nobisi was named by Pichardo (2004) based on a mandible and referred specimens from Valsequillo, central Mexico. Four years after proposing the name, Pichardo (2008) synonymized this species with *E. excelsus*. Although we did not include Pichardo (2008) in our literature survey (because it appeared to be a self-published identification guide without any level of peer review), the fact that he considered *E. nobisi* a junior synonym of *E. excelsus*, makes us question the validity of *E. nobisi*.

We consider *mexicanus*, *idahoensis*, *enormis*, *stenonis anguinus*, *verae*, *cedralensis*, and *pseudaltidens* as potentially valid taxa. The type specimens of *mexicanus*, *enormis*, *stenonis anguinus*, *cedralensis*, and *pseudaltidens* consist of relatively complete cranial and in some cases postcranial remains (Hibbard, 1955; Azzaroli and Voorhies, 1993; Downs and Miller, 1994; Hulbert, 1995; Alberdi et al., 2014). In contrast, the type specimen of *idahoensis* is an isolated upper cheek tooth (Winans, 1985). Nonetheless, large samples of specimens (e.g., Grandview, Idaho, and 111 Ranch, Arizona) have been referred to *idahoensis*, which have distinctive morphological features (Scott, 2005; Barrón-Ortiz et al., 2019) that indicate this is a potentially valid species. The remaining species, *verae*, is known from several cranial and postcranial remains recovered in Alaska and Yukon that are distinctive from other equid remains found in that region (e.g, Harington 1990; 2011).

To summarize, we identify 16 potentially valid species names based on the analysis of 68 studies discussing fossil specimens of North and Central American *Equus* sensu lato. They are: *calobatus*, *cedralensis*, *conversidens*, *cumminsii*, *enormis*, *francisci*, *fraternus*, *idahoensis*, *lambei*, *mexicanus*, *occidentalis*, *pseudaltidens*, *scotti*, *simplicidens*, *stenonis anguinus*, and *verae*.

3.5. Summary of generic and subgeneric names

We did not attempt to conduct a similar analysis to the one described above to study the supraspecific taxonomy of North and Central American *Equus* sensu lato. We do, however, present a summary of the different genera and subgenera to which each of the 16 potentially valid species have been assigned to (Figure S1.4), based on the taxonomic conclusions of the 68 studies we surveyed (i.e., based on the information found in Table S6A). Note that to create these figures, we only used the generic and (if applicable) subgeneric assignments in the studies that considered each species as valid. For example, out of the 20 studies that consider *francisci* as a valid species, it is assigned to *Amerhippus* 1 time, to *Equus* 11 times, to *Equus* (*Asinus*) 2 times, to *Equus* (*Equus*) 1 time, to *Equus* (*Hemionus*) 2 times, to *Equus* (*Onager*) 1 time, and to *Haringtonhippus* 2 times (Table S6A; Figure S1.4).

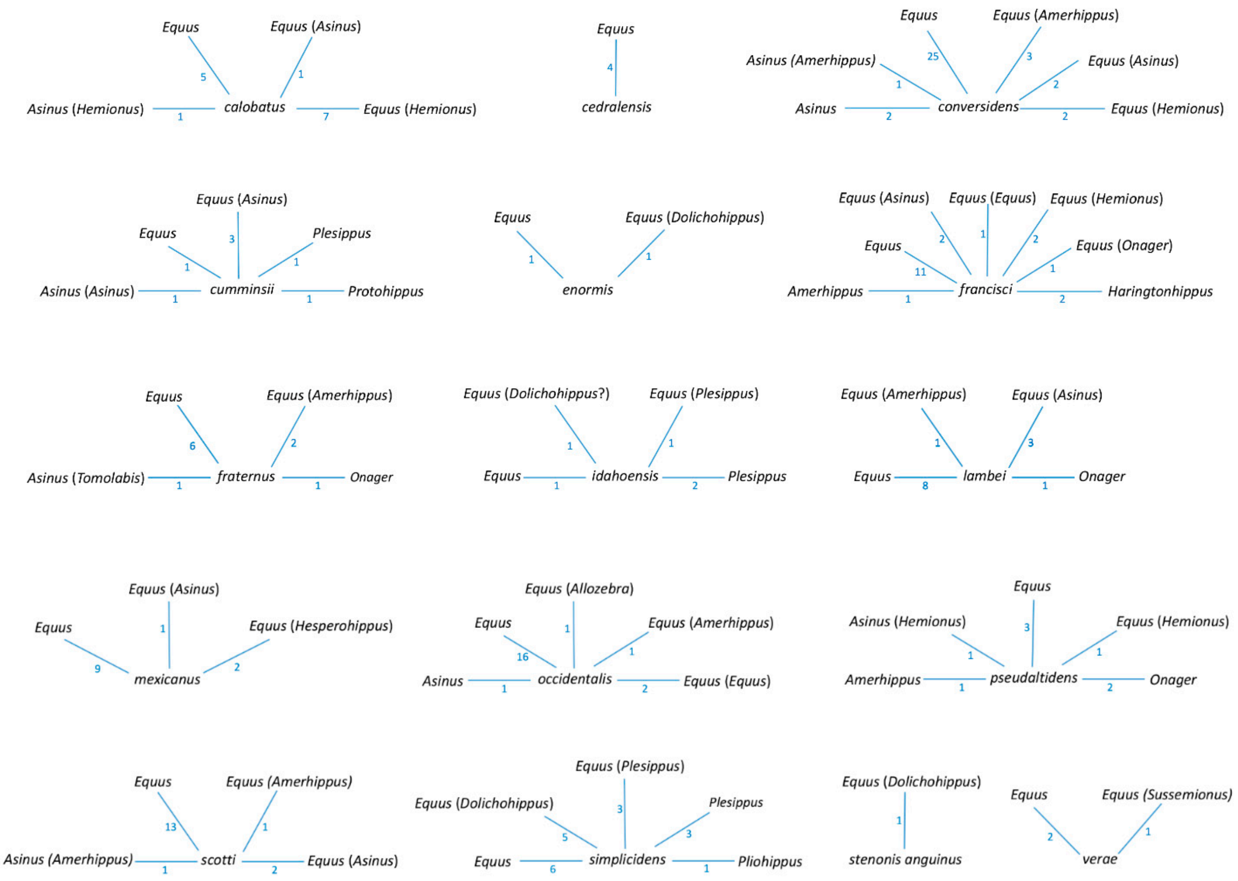

Figure S1.4 Genera and subgenera to which each of the 16 potentially valid equid species have been assigned to based on the studies we evaluated.

3.6. Limitations and assumptions

The objectives of our study were to summarize the taxonomic conclusions of most of the relevant studies discussing fossil specimens of North and Central American *Equus* sensu lato (ranging from 1901 to 2021) and to analyze that information to identify potentially valid species. The primary assumption of our study is that congruence across multiple studies regarding the validity of a particular species indicates that species is likely valid.

The potentially valid species we identified based on the analysis of 68 studies discussing fossil specimens of North and Central American *Equus* sensu lato (ranging from 1901 to 2021) represent taxonomic hypotheses that are testable with further morphological and molecular studies. Our study should not be viewed as a systematic revision of North and Central American species of *Equus* sensu lato. A systematic revision of this group of equids requires a study design that includes direct study of type specimens of all or most named species of North and Central American *Equus* sensu lato and direct study of the most relevant specimen samples referred to each species. We also acknowledge that we used the taxonomic nomenclature that is most commonly used in the literature, without evaluating whether a particular name was the "most correct" name for a given species.

Regarding the generic and subgeneric taxonomy of *Equus* sensu lato, we recognize that this is a topic that has received considerable attention in recent years and different generic and subgeneric names have been used for species of *Equus* sensu lato (e.g., Eisenmann et al., 2008; Eisenmann, 2010; Heintzman et al. 2017; Barrón-Ortiz et al., 2019; Cirilli et al., 2021). Given the diversity of opinions regarding the generic and subgeneric taxonomy of *Equus* sensu lato, we only present a summary of the different genera and

subgenera to which each of the potentially valid species we identified have been assigned to (Figure S1.4). This summary is based on the taxonomic conclusions of the studies we surveyed (i.e., based on the information found in Table S6A).

Despite these limitations, we hope that the information and results we presented will serve as a useful reference for future studies on the evolution and taxonomy of *Equus* sensu lato.

3.7. <u>North and Central America</u>

3.7.1 *Calippus elaschistus* Protostylid reduced on p3-me. Metaflexid and linguaflexid better developed and more persistent with wear than in *Ca. regulus*. Metastyid and hypoconulid not extremely elongated in early wear-stages on m1-m3. Ectoflexid reduced in depth on permanent and deciduous cheek teeth , generally not penetrating ithsmus, except in heavily worn molars. Pli entoflexid absent on molars. Fossettes simple, without plications except in earliest wear-stages. Styles weak, but slightly stronger than those of *Ca. regulus*. Pli caballin absent or vestigial. Hypoconal groove closed with onset of wear, persists as a lake until middle wear-stages on premolars; disappears in very early wera-stages of molars.

3.7.2 *Calippus hondurensis* It may be distinguished by its small size and relatively small protocone.

3.7.3 *Dinohippus leardi* The cheek teeth are long-crowned and moderately curved, with very strong taper toward the root region associated with pronounced changes in the enamel pattern. In the upper teeth the fossettes are simple; a single pli caballin is present. The lower molars are very long and straight, with distinctly separated metaconid and metastylid and prominent external valleys.

3.7.4 *Dinohippus spectans* Crown heights are very long and slightly curved, with short roots short and internal columns (protocone, hypocone) relatively small, subequal in size, and flattened in outline. The fossettes of great transverse width and simple enamel borders, equal to the anteroposterior diameters, crescents correspondingly narrow, one loop (pli caballin) projecting from inner enamel border almost reaching anterior inner column (protocone), a crochet fold projecting into prefossette, and cement is abundant.

3.7.5 *Astrohippus ansae* Described as of size of *Protohippus perditus*, teeth higher crowned and less curved, transverse width relatively less, especially in premolars (in contrast to *P. perditus secundus*); mesostyle much heavier; cement lakes simple without enamel inflections. In the lower teeth the metaconid and metastylis are oval to rounded in outline, the intervening valley narrowed or fading out toward base of tooth, the outer crescents rounded to flattened.

3.7.6 *Astrohippus stockii* The tooth crown of the upper cheek teeth is slightly curved or straight (Lance, 1950). The protocone is relatively large, elongated, with a well-developed anterior projection and a posterior portion that tends to be directed lingually; the protocone often displays a lingual groove (Lance, 1950). The hypoconal groove is usually absent or rudimentary (MacFadden, 1984). The metaconids and metastylids of the lower cheek teeth are relatively small, with parallel lingual and buccal sides and are separate to the base of the tooth crowns (Lance, 1950). The enamel walls of the protoconids and hypoconids are flat (Lance, 1950). The lower deciduous cheek teeth sometimes display protostylids and hypostylids (Lance, 1950). This species also displays a well-developed and relatively large complex of lacrimal and malar fossae (MacFadden, 1984).

3.7.7 *Dinohippus interpolatus* It is distinguished from corresponding teeth of *Protohippus* by the small size of protocone and hypocone. Its protocone unites with protoconule, the pli caballin and crochet fold in prefossette, has a single enamel fold in postfossette, the hypostyle region is simple and fossettes are relatively large with relatively simple borders; crowns are curved transversely, but not anteroposteriorly, and the cement is abundant.

3.7.8 *Dinohippus leydianus* Considered a large size horse with malar fossa absent and monodactyl metapodials. Dorsal preorbital fossa poorly developed, rudimentary, or absent; moderately curved upper dentition (relative to *Pliohippus*). The protocone is strongly connected to the proselene, with well-developed hypoconal groove and simple enamel plications. The lower dentition has relatively deep ectoflexids.

3.7.9 *Dinohippus mexicanus* Nasal notch retracted to a position dorsal to the P2. Moderately hypsodont. Premolar and molars are moderately curved transversely. Dorsal preorbital fossa moderately well developed dorsally, ventrally, and posteriorly, although characteristically lacking a distinct rim. Long preorbital bar between DPOF and orbit. Malar fossa very poorly developed, or absent. Cheek tooth enamel pattern generally simple. Upper cheek tooth protocones oval and moderately elongated, especially posterior to the connection with the protoloph, and pre- and postfossettes crescentic in occlusal cross section with few plications. Hypoconal groove well developed and persists until late wear. Lower premolars and molar with metaconids and metastylids have rounded border and are well-separated ectotoflexids modetately deep in the premolars and deep in the the molars, and pli caballinids poorly developed or absent.

3.7.10 *Cormohipparion occidentale* Protocons are oval and relatively elongated. Fossette bordes most plicated of all North American hipparions. In lower premolars, shallow ectoflexids and moderately developed plica caballinids and in molar deep ectoflexids and pli caballinids rudimentary or absent. Protostylids are well developed or sometimes isolated. Metaconids, metastylids, and entoconids are usually elongated, expanded and large. Hypoconulids with rounded or angular borders.

3.7.11 *Nannippus aztecus* Protocone isolated nearly to the base of crown in P3 to M3 and attached by a narrow isthmus in extreme wear. Protocones are relatively compressed and elongated, with long axis oriented lingually, especially in well-worn teeth. Enamel fossettes are much plicated.

3.7.12 *Nannippus lenticularis* Upper molar teeth have a very distinct form of the protocone, which is long separate from the paraconule. It has an absolutely lenticular section, presenting acute angles anteriorly and posteriorly, and convex surface internally and externally.

3.7.13 *Nannippus peninsulatus* As with other species of the genus *Nannippus*, *N. peninsulatus* lacks facial fossae and has an elongate rostrum (MacFadden, 1984). It differs from other species of *Nannippus* in having higher crowned teeth, very slender metapodials, and in the absence or extremely rudimentary nature of the protostylid (MacFadden, 1984).

3.7.14 *Neohipparion eurystyle* The dorsal preorbital fossa is absent. Protocone very elongated anterior posteriorly and usually with angular anterior and posterior borders. Premolars and molars are relatively straight transversely. Premolars generally with prominent expanded and flattened parastyles. Fossettes in upper teeth moderately to very plicated. Pli caballin of multiple loops. Lower teeth very shallow ectoflexids, well-developed plia caballinds, separated metaconids and metastylids, elongated entoconids and hypoconulids, and prominent isthmuses.

3.7.15 *Neohipparion leptode* Dorsal preorbital fossa absent. Teeth are relatively hypsodont. Relatively elongated protocones. Fossette borders are moderately complicated. Pli caballinids well developed in the premolars but less well developed in the molars. Metaconids, metastylids, entoconids, and hypoconulids moderately expanded with rounded or angular enamel borders.

3.7.16 Hipparionini genus and species indeterminate The specimen consists of associated maxillae and premaxillae with the right DI1 and DP2-DP4 and the left DP1-DP4 of a young foal (approximately 6 - 10 months in age) (Hulbert and Harington, 1999). It is a relatively large hipparionine equid (estimated adult tooth row length of 150 mm), with deciduous premolars that have low crowns, complex enamel plications, and oval,

isolated protocones, and a facial region that shows a reduced preorbital fossa located posterior to the infraorbital foramen (Hulbert and Harington, 1999).

3.7.17 *Neohipparion gidley* Highly hypsodont teeth with very elongated protocones, elongated and flattened parastyles and mesostyles. The pli caballins are well developed. The lower teeth have very expanded conids and stylids with pli caballinids well developed, shallow ectoflexids and well-developed pli- cations on the isthmuses.

3.7.18 *Boreohippidion galushai* It has the nasal notch retracted to above P3-P4 and a fossa between buccinator and lacrimal fossa not as well developed as other *Onohippidium*.

3.7.19 *Cormohipparium emsliei* The taxon exhibits an extremely complex fossette pli- cations and better developed, more persistent pli caballinids in relation to all North American species of *Cormohipparion*. Large unworn molars. Metaconid and metastylid of dp2-dp4 and p2-p4 with unique lingual groove in early wear-stages.

3.7.20 *Pseudohipparion simpsoni* Incisors, premolars and molar incipiently hypselodont; cheek teeth smaller than in any other species of *Pseudhipparion*; relatively simple enamel patterns, fossettes absent in late wear stages; large protocone connects with protoloph in middle to late wear stages; in premolars the hypoconal groove closes to form a fossette in early wear stages and is lost in mid to late wear stages, in molars the hypoconal groove persists until latest wear stages. Metaconid and metastylid elongate and curved lingually; protostylid greatly reduced or lost; lower deciduous premolars lack ectostylids and are very high-crowned.

3.7.21 *Pliohippus coalingensis* It is distinguished by its large size, unusual narrowness of cheek-teeth in transverse diameter, small protocone and narrow, simple fossetes.

3.7.22 *Nannippus beckensis* Mean cheek tooth row lenght ca, 102 mm. Unworn or little-worn mesostyle crown height of M1 ca. 47-53 mm. Dorsal preorbital fossa is absent. Protocone is oval with rounded borders. Fossette borders are relatively simple to moderately plicated. Protostylids are usually absent. Ectoflexids are moderate to deep. Pli caballinids poorly developed, rudimentary, or absent (MacFadden, 1984).

3.7.23 *Equus simplicidens* It is characterized by a long skull with prominent occipital region, slender limb bones, relatively simple enamel pattern on cheek teeth and presenting enamel foldings on the lower incisors with narrow, not flattened, meta- and paracones (Cope, 1892; Kurten and Anderson, 1980). It presents a relatively short rostrum, a shallow preorbital fossa, shallow position of the narial notch at the suture and, broadly triangular hamuli and a narrow basisphenoid and basioccipital (Downs and Miller, 1994; Skinner, 1972) with limb bones overlapping with *E. enormis* and *E. stenonis anguinus* (Azzaroli, 1995). Hagerman horses were described as presenting a shallow facial fossa, long muzzle and face, orbit set relatively back, greater downward inflection of the basicranial region, greater curvature of the cheek teeth, simpler fossettes, and shorter protocone (Winans, 1985). The revised diagnosis for the Hagerman horses made by Winans (1985) consider it a large horse, with basicranial length greater than 50cm, not stilt-legged (mc3 length/proximal width ratio less than 5; mt3 length/proximal width less than 6), the ectoflexid penetrates the isthmus, P1 is frequently retained and lies directly anteriorly to the anterior end of P2. A quantitative review of North American *Equus* (Winans, 1989) revealed an *E. simplicidens* group, including *E. pons*, *E. shoshonensis* and *E. simplicidens* with the following description: a large stout legged horse, slightly larger than *E. scotti* group, with elongated and narrow rostrum, but not wider at the anterior end than across the diastema. It has primitive *Pliohippus*-like characters seen in its nearly circular protocones, distinct facial fossa on skull, deep ectoflexid and broadly V-shaped linguaflexid (Winans, 1989).

3.7.24 *Equus idahoensis* It is diagnosed by a large tooth, protocone short anteroposteriorly with a concave lingual border, narrow and V-shaped double-knot (Winans, 1985).

3.7.25 *Equus enormis* The skull is large. The basicranial length is greater than 660 mm on the available material. Adult upper and lower third incisor massive; lower third incisor without infundibulum and with a V-shaped linguaflexid. Molar ectoflexids enter but do

not fully penetrate isthmus, metastylid in p3 and m1-m2 broad transversely, triangular to rounded anteriorly. Mc3s, Mt3s, and phalanx are large and robust, resembling *E. simplicidens*.

3.7.26 *Equus cumminsii* The upper cheek teeth have simple enamel, short protocones, and a very simple enamel pattern in the inner fossettes (Forsten, 1992; Azzaroli, 1990; Azzaroli and Voorhies, 1993). It presents similar sizes to *E. semiplicatus* and *E. tau* but is distinguished by a greater concavity of the labial borders of the pre and postfossettes (Winans, 1985).

3.7.27. *Equus calobatus* It is potentially distinguished from other North American stilt-legged species by its larger size and very long molar entoflexids (Hulbert, 1995).

3.7.28 *Equus scotti* It is considered a large not stilt-legged horse (mc3 length/proximal width ratio less than 5; mt3 length/proximal width less than 6) with basicranial length greater than 50cm (Winans, 1985). Its ectoflexid penetrates isthmus on m1 and m2, but not usually on m3, and while the P1 is frequently retained, it generally lies slightly interior to the anterior end of P2 rather than anterior to P2 (Winans, 1985). A quantitative analysis for the *E. scotti* species group (including *E. hatcheri*, *E. niobrarensis*, and *E. scotti*) made by Winans (1989) described it as comprised of large stout-legged horses with basicranial length greater than 50cm and wider rostrum in proportion to skull length.

3.7.29 *Equus stenonis anguinus* There is also a deep furrow along the digital nasal suture. Regarding the upper cheek teeth, the protocones are slightly more elongated and lingually grooved than in the European *E. stenonis*, but falling within the range of variation of specimens from the type area.

3.7.30 *Equus conversidens* Azzaroli [70] described a fragmentary skull (LACM 308/123900) from Barranca del Muerto near Tequixquiac, Mexico, in which the "two tooth rows converge rostrally, giving evidence that the palate of the holotype (of *E. conversidens*) was correctly mounted and that Owen's name is after all appropriate."

3.7.31 *Equus lambei* They are smaller-bodied horses with broad skulls, and the lower profile of their mandible rises anteriorly relative to the cheek teeth. The cheek teeth are generally caballoid in morphology, with a wide U-shaped linguaflexid groove, and the protocones are unusually long. When compared to the domestic horse (*E. caballus*), *E. lambei* has a less prominent maxillary ridge and a more strongly vaulted palate. The space between the orbit and the infraorbital foramen is also convex in *E. lambei*, not concave as it is in the domestic horse. The metapodials of *E. lambei* are slenderer than those of the Przewalskii's horse, but are more robust than those of hemiones and *Equus francisci*.

3.7.32 *Equus* (or *Haringtonhippus*) *francisci* A revised diagnosis by Winans (1985) characterized it as small stilt-legged horse (mc3 length/proximal width ratio greater than 5; mt3 length/proximal width ratio greater than 6), with basicranial length less than 50cm, ectoflexid that does not penetrate isthmus and P1 usually not retained. Winans' (1989) quantitative description for her *E. francisci* species group (including *E. onager altidens*, *E. onager arellanoi*, *E.* (*Asinus*) *calobatus*, *E. francisci*, *E. quinni*, *E. zoytalis*) considered it a stilt-legged horse with greater metapodial size range than other groups and skulls more elongated and narrower than average.

3.7.33 *Equus fraternus* It was diagnosed as a relatively small sized equid, as in *E. conversidens* and *E. francisci*, small protocone and no infundibulum (Winans, 1985).

3.7.34 *Equus pseudaltidens* Upper P2 and P3 show hypoconal fossettes (Quinn, 1957; Hulbert, 1995). Lower i1 and the di2 lack enamel cups and the lower p2 shows a pli protostylid (Quinn, 1957; Hulbert, 1995; Eisenmann et al., 2008).

3.7.35 *Equus verae* There was an almost complete skeleton of *E. verae* found in the same exposure 21 locality (collection 835 GIN), including upper 2xP4, M1, M2, upper incisors, lower premolars, numerous postcranial bones (femurs, astragali, metatarsals, phalanxes)

(Sher, 1971). The holotype is only the mandible because although morphologically same the rest of teeth and bones are not necessarily from a single individual. Described by Sher (1971) the holotype teeth row is larger 216 mm and high-crowned, a double knot with a rounded metaconid on molars and slightly angular in premolars, and triangular mesostylids, both noncaballoid patterns ("unparallel walls" in metaconid and narrow entry in mesostylid); a linguaflexid "V" shaped in premolars with an angle 120-130°, and a milder "V" with an angle 110° in molars, very deep ectoflexid in molars, more shallow in premolars; rectangular entoconid, long diastema, narrow incisor area. The protocone is short and situated posteriorly to the narrow isthmus, with the premolar protocone length was 33-39% of the tooth length, molar protocone – 33.4-43.5%; the hypocone is situated more inside the tooth, the mesostyle is "cloven", the M3 has a deep groove through the whole height of the tooth.

3.7.36 *Equus occidentalis* The lectotype specimen is characterized by a short triangular protocone and a simple enamel pattern; because the specimen is a single tooth, no other characters are available. Referred specimens from Rancho La Brea represent a large equid with stout limbs, a broad muzzle, domed frontals, short triangular protocones, U-shaped linguaflexids, nonpenetrating molar ectoflexids, and a complete lack of infundibula in the lower incisors.

3.7.37 *Equus cedralensis* The upper teeth have a protocone that is elongated more distally than mesially and with a small lingual groove; with styles, parastyle, and mesostyle on premolars that are wide with a groove and narrow without grooves on molars. The lower teeth have metaconid-metastylid (double knot), rounded or slightly angular, respectively. The ectoflexid is superficial in premolars and molars without crossing the isthmus, only on molars with an advanced wear the ectoflexid is deeper, crosses the isthmus, and could touch the linguaflexid; the linguaflexid is large and more or less U shape. The metapodials of this species are relatively long and slender, with phalanges slightly large and narrow in the diaphysis and distal articulation (Marín-Leyva et al., 2019).

3.7.38 *Equus mexicanus* The cranium is large; basicranial length is greater than 500 mm at the available material. The upper cheek teeth are large, with elongate flattened protocones, derived features, and average complexly plicate enamel. Lower molars in referred specimens exhibit shallow ectoflexids on premolars, penetrating the isthmus on m1, m2, and m3 only in advanced wear. Referred metapodials of this horse are long but robust, and so it is not interpreted to be a stilt-legged horse (mc3 length/ proximal width ratio less than 5; mt3 length/proximal width less than 6); referred phalanges are compact, robust, and broad in the distal articulation (Marín-Leyva et al., 2019).

3.8. South America

3.8.1. *Equus neogeus* It is characterized by the lack of infundibula in the lower incisors and a continuum of gradual linear variation in its autopodia (Hoffstetter, 1950; Machado et al., 2017; Machado and Avilla, 2019). The autopodia intraspecific variation, highlighted in the metapodials, seems to be related to topographic relief and is postulated that *E. neogeus* represents a smooth cline of phenotypic variation (Machado et al. 2017; Machado and Avilla, 2019).

3.8.2. *Hippidion saldiasi* The nasal notch is retracted to M3 and it is considered the smallest of the *Hippidion* (Prado and Alberdi, 2017).

3.8.3. *Hippidion principale* It is characterized by variations in the nasal notch relative to presence or absence of the dorsal preorbital fossa (DPOF): when present, the nasal notch is placed at M3 or behind it, and skulls without it have the nasal notch at the level of M2 (Prado and Alberdi, 2017).

3.8.4. *Hippidion devillei* The nasal notch is retracted to the level of M2 and is considered an intermediate slender middle-sized *Hippidion* in comparison with *H. principale* and *H. saldiasi* (Prado and Alberdi, 2017).

4. Systematics of the Equinae since 5.3 Ma in Eurasia and Africa

4.1. Eastern Asia (Central Asia, China, Mongolia and Russia)

4.1.1. *Plesiohipparion houfenense* A large hipparion with shallow nasal notch, maxillary incisors grooved, cheek teeth high crowned (60-70 mm) and with elongate protocones. Mandibular cheek teeth with pointed metaconids and metastylids. Limb bones are elongate and robust.

4.1.2. *Proboscidipparion pater* This species is remarkable for its strongly retracted nasals accompanined by a dorsal convexly arched premaxillary region. This species lacks a preorbital fossa. Cheek teeth are high crowned (60-80 mm) with tooth row being 140-180 mm in length. Maxillary cheek teeth complexly plicated, protocone small and oval. Mandibular cheek teeth with angular metaconids and metastylids and wide linguaflexid (Qiu et al., 1987).

4.1.3. *Plesiohipparion huangheense* The lower cheek teeth are similar to *P. houfenense* but with very great compression and pointing of metaconid and metastylid and corresponding wide and deep linguflexid.

4.1.4. *Cremohipparion licenti* A small hipparion with multiple facial fossae, very short preorbital bar and highly retracted nasals with nasal notch retracted to M1 mesostyle. Maxillary cheek teeth are small, with simply plicated fossettes and rounded protocone. Cheek tooth row 120-135 mm.

4.1.5. *Baryhipparion insperatum* It is a large enigmatic hipparion with very long POF, nasal notch shallow extending anterior to P2, maxillary cheek teeth are low crowned, not exceeding 50 mm., fossettes with fine and short plications; metaconid-metastylid can be angular. *Baryhipparion* has an intriguing constellation of advanced and primitive traits.

4.1.6. *Plesiohipparion shanxiense* It is most similar to the younger species *P. houfenense* differing in its larger size, longer snout, length of the cheek tooth row. As in *P. houfenense*, it lacks a preorbital fossa altogether and the nasal notch is retracted only anterior to P2. Maxillary P2 is moderately elongate, protocones are elongate and triangular with lingual flattening; pre- and postfossettes are moderately richly plicated; pli caballins vary from single to double. Mandibular cheek teeth have labiolingually restricted pre- and postflexids; metaconids and metastylids are mostly angular and pointed lingually with broad, deep linguaflexids.

4.1.7. *Proboscidipparion sinense* This species has even more retracted nasals coming to the level just anterior to the orbits.

4.1.8. *Equus eisenmannae* Following the last emended diagnosis of Sun and Deng (2019), *E. eisenmannae* is characterized by its large sized cranium, and an elongated snout; the preorbital fossa is shallow and placed high in the maxilla and a long distance between orbit and third molar. The lower cheek teeth have rounded metaconids and metastylids and shallow linguaflexids on lower molars. The postcranial elements are large and elongated, with the dimension similar to *E. livenzovensis*, whereas the cranial proportions are similar to those of *E. simplicidens* than *E. stenonis* (Cirilli et al., 2021a, 2021b).

4.1.9. *Equus sanmeniensis* The cranium is elongated, with a basal morphology close to *E. simplicidens* and with an elongated snout. The POF is large and placed high in the maxilla, whereas the orbit is placed well behind the third molar. The protocone is elongated and concave lingually. The lower cheek teeth have V-shaped linguaflexids with a deep ectoflexid.

4.1.10. *Equus huanghoensis* The cranium is large with an elongated snout, with an undulated outline in lateral view. The nasal notch extends to boundary of P3/P4. The POF is placed high in the maxilla and well developed, although with an idefined lower margin. The orbit is placed well behind of the third molar. There are short protocones, poorly developed pli caballin and simple pre- and postfossettes. The mandibular cheek teeth have the V-shaped linguaflexid.

4.1.11. *Equus yunnanensis* The latest description of Sun and Deng (2019) reports a relatively large cranium, with an elongated snout. The upper cheek teeth are medium sized with a short protocone have a medium sized, with a V-shaped linguaflexid, rounded metaconid and metastylid, and shallow ectoflexid in mandibular cheek teeth. The distal limbs are robustly built.

4.1.12. *Equus teilhardi* The cranial morphology shows a nasal notch extending above the P2/P3 boundary, a short snout, a shallow preorbital fossa with indistinct border, the lateral cranial outline is undulated and a deep groove along the nasal suture. The maxillary cheek teeth have simple fossettes, short protocones and small pli caballins. The lower cheek teeth have V-shaped linguaflexids with deep ectoflexids on molars. The limb bones are short and robust.

4.1.13. *Equus qyingyangensis* It is characterized by an extension of the nasal notch between P2/P3, the preorbital fossa is shallow with indistinct border and deep groove along the nasal suture. The upper teeth have long protocones and weak a pli caballin. The lower teeth have typical V-shaped linguaflexids and deep ectoflexids up to the linguaflexids on the lower molars. The limb bones are slender. Sun and Deng (2019) recognized a close similarity between *E. qyingyangensis* and *E. sanmeniensis*, although with some discrete differences in cranial morphology (e.g. the distance between the temporal condyle and the posterior margin of the orbital bar). Nevertheless, *E. qyingyangensis* has slender third metapodials, similar in size and morphology to *E. simplicidens*.

4.1.14. *Equus wangi* This species shows some dental similarities with *E. eisenmannae* (e.g. large cheek teeth, short pli caballin, simple mesostyle, strong enamel plication, rounded double-knot, shallow ectoflexid and elongated postflexid) and to *E. yunnanensis* (circular metastylids and shallow ectoflexids).

4.1.15. *Equus pamirensis* The species is known from Kuruksai (Kazakhstan). The sample include some deformed skulls and postcranial elements. The skulls has the nasal notch retracted up to the P3 mesostyle.. The POF is faintly delimitated and placed high on the maxilla, and the oribit is placed behind the posterior border of the M3. In ventral view, the palate is large, and the choanae extend up to the M2/M3 boarder. The upper dentition has a short protocone, as in *E. stenonis*, whereas the lower dentiton has a V-shaped linguaflexid. Third metapodials are large and robust.

4.1.17. *Equus* (*Hemionus*) *nalaikhaensis* The skull is almost complete even damaged, lacking the upper cheek tooth rows (few teeth are preserved). It shows a slender morphology with an undulated forehead profile in lateral view. The nasal notch extend above the P2. The POF is faintly delimitated and placed high on the maxilla, and the oribit is placed above the posterior border of the M3. In ventral view, the palate is slender, and the choanae extend up to the M2/M3 boarder (Eisenmann and Kuznetsova 2004).

4.1.18. *Equus coliemensis* The skull of *Equus coliemensis* has a broad forehead with elongated snout and narrow supra-occipital crest, rather small teeth with triangular protocone (Lazarev, 1980). The length of the skull 542.8mm, molar row is 180.1 mm long (Kuzmina, 1997). Kuzmina (1997) suggested that all varaitions in teeth plications and the length of metapodia lies within individual variation of *Equus suessenbornensis*.

4.1.19 *Equus lenensis* The Lena horse or the horse of Cherskogo was small, around 1.3-1.4m high at the withers (Kuzmina, 1997). The small skull of *Equus lenensis* is charterized by a broad forehead and the upper teeth with a long protocone (Lazarev, 2008).

Compared to *Equus przewalski* the Lena horse's skull was longer (465-503.6mm), wider and taller but the teeth row is shorter (Kuzmina, 1997).

4.1.20. *Equus beijingensis* The *E. beijingensis* maxillary and mandibular cheek teeth morphology shows a long protocone, moderately plicated pre- and postfossettes and the U-shaped linguaflexid.

4.1.21. *Equus valeriani* Little is known about the morphology of this species, most material is cited by Eisenmann et al. (2002). This taxon is characterized as having a long protocone with a small notch on its lingual side, and a primitive symmetric shape of the double knot in lower premolars. Gromova (1946, 1949) regards *E. valeriani* as an unusual horse with a mixture of advanced caballine and primitive stenonine dental characters: it is not approached by *E. hemionus* and *E. hydruntinus* for its large body size, but shows some morphological differences from *E. ferus* and *E. przewalskii* (e.g. the stenonine shape of the metaconid-metastylid complex). Gromova 1946 also considered *E. valeriani* close to *E. sivalensis.*

4.1.22. *Equus dalianensis* Poor is known about the morphology of this species, but a rencent work on its mitochondrial genome identify it as a separate clade in the caballine horses (Yuan et al. 2020).

4.1.23. *Equus ovodovi* According to Eisenmann and Vasiliev, (2011) *Equus ovodovi* is characterized as being a slender-limbed *Equus* slightly larger than the extant *E. hemionus onager*. The cheek teeth are relatively small, with a shallow postprotoconal grooves on the upper cheek teeth, shallow lingual grooves, elongated double knots and metaconids on the lower cheek teeth. Deep vestibular grooves on the mandibular molars and occasional presence of a pli protostylid on p2.

4.1.24. *Equus hemionus* The skull has a medium size, with an amost linear forehead profile. The muzzle is short, and the nasal notch extend above the P2. The maxillary crest is developed up to the P4/M1 boarder, whereas the POF is almost oblitered. The orbit is placed above the posterior boreder of the M3, and the braincase is deflected and relatively small. The upper dentirion shows an elongated, longer in the molars than premolars, a large and squared mesostyle and a round hypocone. The lower dentiton has a V-shaped linguaflexid, with the metastylid pointed on the lingual side. The ectoflexid is developed preflexid and the postflexid. Pli caballinid present on the hypoconid. Third metapodials are medium sized and slender.

4.1.25. *Equus kiang* This species has a cranial morphology similar to *E. hemionus*. The skull has a medium size, with an amost linear forehead profile. The muzzle is short, and the nasal notch extend above the P2 mestostyle. The maxillary crest is developed up to the P4/M1 boarder, whereas the POF is faintly delimited. The infraorbital foramen is placed above the P4 mesostyle. The orbit is placed above the posterior boreder of the M3, and the braincase is deflected and relatively small. The upper dentition shows an elongated, longer in the molars than premolars, a large and squared mesostyle and a round hypocone. The lower dentiton has a V-shaped linguaflexid, with the metastylid pointed on the lingual side. The ectoflexid is not well developed. Third metapodials are medium sized and slender.

4.1.26. *Equus przewalskii* The skull is elongated, with an amost linear forehead profile. The muzzle is long, and the nasal notch extend above the P2 mestostyle. The maxillary crest is developed up to the P4/M1 boarder, whereas the POF is almost oblitered. The infraorbital foramen is placed high above the P4 mesostyle. The orbit is placed above the posterior boreder of the M3, and the braincase is deflected and elongated. The upper dentition shows an elongated and asymmentrical protocone, longer in the molars than premolars, a large and squared mesostyle and a pointed hypocone. The lower dentiton has a U-shaped linguaflexid, with the metastylid pointed on the lingual side. The ectoflexid is more elongated in molars than premolars. Third metapodials are large and roboust.

4.2. Indian Subcontinent

4.2.2. *Plesiohipparion huangheense* The specimen has elongated and lingually pointed metaconid and metastylids with an accompanying broad, U-shaped linguaflexid characteristic of *P. huangheense*.

4.2.3. *Eurygnathohippus* sp. A maxillary P3/P4 from the same locality as WIF/A 124 a mandibular m1, was also referred to this taxon, but because upper cheek teeth do not possess any diagnostic characteristics for this genus, Jukar, et al. (2019) used size comparisons with the Ethiopian taxon *Eurygnathohippus hasumense* to refer the specimen to the genus.

4.2.4. *Equus sivalensis* The preorbital fossa is weakly developed, protocones are weakly elongated on P4-M1 but longer on M2-M3. Hypoglyphs are moderately incised, fossettes are moderately ornamented. The mandibular premolars have elongated metaconids and squared metastylids. Linguaflexids are narrow and v-shaped and ectoflexids are not very deep, not invading the space between pre and post flexids. This species is known from a number of crania, mandibles, and postcrania housed in museums in India, Europe, and North America (Bernor, et al. 2019).

4.2.5. *Equus* sp. A small species of *Equus* with smaller slender metapodials has been reported from the Pinjor Formation of the Upper Siwaliks.

4.2.6. *Equus namadicus* NHMUK PV M.2683 is a skull of an old individual. Pre-orbital fossa is large and shallow. Nasal notch extends to the P3. Dentition is similar to *E. sivalensis*, but protocones are more elongated on both premolars and molars. E. namadicus, like *E. sivalensis* has broad zygomatic arches.

4.3. Europe

4.3.1. *Plesiohipparion longipes* The taxon is characterize by its elongated and slender third metapodials and proximal phalanges. Koufos and Vlachou (2005) reported a maxilla from a young adult from Akkasdagi showing an elliptical and lingually flattened protocone, with a "spur" in the P2. The pli caballin is strong, double in the premolars and simple in the molars. The hypocone is elongated and angular with deep and open distal hypoconal groove.

4.3.2. *Cremohipparion fissurae* The maxillary cheek teeth from Layna (Spain) have moderately complex plications, weakly developed pli caballins, and protocones that are triangular and oval to lingually flattened. The mandibular cheek teeth have lingually pointed metaconids and metastylids, and mandibular lower third molar has a distally extended talonid. The La Gloria 4 (Spain) mt3 is very elongate and slender.

4.3.3. *Proboscidipparion crassum* The taxon is characterized by a complicated enamel morphology of the upper cheek tooth rows, with a short and lingually flattened protocone, with a ramified pli caballing and wide hypocone. The lower cheek teeth shows a rounded metaconids and metastylids, with a deep ectoflexis in premolars and molars. Third metapodials and proximal phalanges are short and roboust.

4.3.4. *Proboscidipparion heintzi* The juvenile cranium has cheek teeth with round and short protocones, hypoglyphs moderately deeply incised and dP1 is worn but large and appearing functional. There is also a juvenile mandible with dp2-4 have V-shaped and shallow linguaflexids; metaconids and metastylids are rounded. An i1 exposed within its crypt of this individual is very large. Bernor and Sen (2017) associated this skull with the holotype mc2, mc3 and mc4 proximal limb which has a short and robust morphology.

4.3.5. *Plesiohipparion rocinantis* The cranium shows a small a preorbital fossa, maxillary cheek teeth with moderately complex plications, small and simple pli caballins, and lingually flattened and labially rounded protocones. The mandibular cheek teeth have pointed metaconids and metastylids with deep, broadly U-shaped linguaflexids. Third

metapodials and first phalanges of the central digit are elongated and slender. These are all morphological hallmarks of *Plesiohipparion*.

4.3.6. *Equus livenzovensis* The cranium is large and elongated with estimated basal length between 600 and 640 mm, with a transversal undulated forehead, a POF faintly delimited and placed high on the face, a well–developed maxillary crest and a strongly deflected braincase. Maxillary cheek teeth show a triangular–shaped, a short protocone increasing in length serially from M1–M3. Mandibular cheek teeth have the typical stenonine V–shaped linguaflexid separating metaconid and metastylid. Limb bones robustly built and the same size of *E. eisenmannae*; longer than in *E. stenonis*, but smaller than in *E. major* and *E. suessenbornensis*; metapodials elongated, and robustly built.

4.3.7. *Equus major* It has stenonine dental features including a protocone serially longer from P2 to M3, a developed pli caballin, and. Mandibular cheek teeth with the typically stenonine V–shaped linguaflexid. Upper and lower cheek teeth have a generally more complicated enamel morphology. Third metapodials are elongate, stout and massive; longer and larger than *E. livenzovensis* and *E. suessenbornensis*.

4.3.8. *Equus stenonis* The taxon has a large-sized monodactyl horse, with a large and elongated cranium with an undulated forehead profile in lateral view and with a deep incision of the nasal notch extending caudally from above the maxillary P3 mesostyle. The POF is faintly delimitated and placed high on the maxilla extending posteriorly to the lacrimal bone. The upper cheek have a triangular, short protocone serially longer from M1 to M3. The lower cheek teeth with the typical stenonine V–shaped linguaflexid separating metaconid–metastylid, with the rmetaconid being rounded and metastylid angular and pointed lingually; ectoflexid well developed and deeper in lower molars than premolars. Third metapodials elongate and robustly built.

4.3.9. *Equus senezensis* The cranium is reduced in length and but large, with a deep incision of the nasal notch extending caudally above the maxillary P3 mesostyle. The braincase is small and flexed downward. The POF is faintly delimited located high on the face and placed posteriorly, close to the lacrimal bone. Maxillary cheek teeth with the stenonine triangular and short protocone, which becomes longer serially from M1 to M3. Premolars and molars reduced more than in *E. stenonis*, but larger than *E. stehlini*. Mandibular cheek teeth with the typically stenonine V–shaped linguaflexid separating the rounded metaconid from the distolingually pointed metastylid. Pli caballinid developed on the labial margin of the hypoconid. Robust metapodials less elongated than *E. stenonis*, but larger than *E. stehlini*.

4.3.10. *Equus stehlini* The cranium is small and slender, with a deep incision of the nasal notch extending caudally above the maxillary P3 mesostyle; braincase is small and flexed downward; faintly delimited POF located high on the face and placed posteriorly, close to the lacrimal bone. Maxillary cheek teeth with a typically stenonine triangular and short protocones, which becomes longer serially from M1 to M3. Mandibular cheek teeth have the stenonine V–shaped linguaflexid separating the rounded metaconid from the distolingually pointed metastylid. Short and robust third metapodials.

4.3.11. *Equus altidens.* This species has a moderate size and slender cranium with the nasal notch retracted to P2 mesostyle. The preorbital fossa is shallow with no peripheral rim and placed high on the maxilla. The infraorbital foramen is placed above P4 mesostyle and above the anteriormost end of the facial–maxillary crest. Cheek tooth morphology is typically stenonine with maxillary protocone being triangular shaped and lingually flattened, the postprotoconal groove is somewhat deep, the hypoglyph moderately incised, and the pli caballin sometimes obliterated in worn maxillary cheek teeth. Mandibular cheek teeth consistently have a V–shaped linguaflexid separating a rounded metaconid and squared and often lingually pointed metastylid; molar ectoflexids very deeply incised. The metapodials and phalanges are more elongate and slenderer than in other stenonine species, including *E. senezensis*.

4.3.12. *Equus suessenbornensis* The V–shaped linguaflexid of the lower cheek teeth defines this species as being a member of the stenonine group. It is larger than *E. livenzovensis* and *E. stenonis* but smaller than *E. major*. No skulls are known. The maxillary and mandibular cheek teeth exhibit a complicated and highly ornamented morphology, as a complex ornamentation of the pre– and postfossettes, an elongated pli caballin and a pointed hypocone. Protocones are large with a squared protocone becoming longer serially from P2 to M3, and with concave lingual margins. Mandibular cheek teeth consistently have a V–shaped linguaflexid separating a rounded metaconid and squared and lingually pointed metastylid; with elongated pli caballinids. Large and massive third metapodials and phalanges.

4.3.13. *Equus apolloniensis* Following Gkeme et al. (2021), *E. apolloniensis* shows an elongated cranium with a short and wide muzzle. The nasal notch extends up to the P2/P3 boarder, the POF is restricted due the high hypsodonty, and the infraorbital foramen is placed above the P3/P4. The maxillary crest extends up to the P4/M1, whereas the orbit is placed well behind the M3. In ventral view, the palatine process and the palate is large and wide, with a circular incisor arcade. It shows a short protocone in P2, which becomes serially more elongate in premolars and molars. The lower dentition shows a V-shaped linguaflexid, with a pointed metastylid. The ectoflexid is short and not well developed. Third metapodials are elongated and large.

4.3.14. *Equus wuesti* The holotype is comprised by two fragmentary mandibles with p2-m3, in a late stage-of-wear. The occlusal morphology is amost oblitered, even showing a V-shaped and short linguaflexid, with a pointed and squared metaconid. The postcranial elements, mostly third metapodials and proximal phalanges are elongated and slender. Overall, all these characters reseamble the European *E. altidens* populations. Moreover, the range of measurements given by Musil (2001) are included in the range of variability shown by *E. altidens* from Sussenborn.

4.3.15. *Equus petralonensis* Following the latest morphological remarks by Koufos et al. (2022), *E. petraloniensis* is characterized by its gracile and slender metapodials. The lower teeth are typical stenonoid (V-shaped ectoflexid with variable depth).

4.3.16. *Equus graziosii* The cranium is wide but not elongated, missing part of the brain case. The muzzle is slender, with the narial notch extending above the P2. The infraorbital foramen is placed above the P4 mesostyle, while the maxillary crest extends up the P4/M1 boarder. The orbit is placed above the posterior boarder of the M3. The POF is poorly represented.

4.3.17. *Equus hydruntinus* The cranium figured in Van Asperen et al. (2012) is short and large, with an undulated forehead profile and a short muzzle. The nasal notch is placed above the P2, the maxillary crest extends up to the P4/M1, whereas the orbit is large and placed above the posterior boarder of the M3. The POF is present, even is faintly deliminted. The POB is short. The braincase is reduced. The upper cheek teeth show a short protocone, whereas in the lower dentition has a V-shaped linguaflexid. Metapodials are short and slender. Overall, its morphology reasembles that of an Asian ass.

4.3.18. *Equus ferus* The cranium is elongated and large, with and undulated forehead profile. The nasal notch extends above the mesostyle of the posterior border of the P2, whereas the infraorbital foramen is placed high above the mesostyle of the P4. The maxillary crest is strong and extends above the P4/M1, whereas the POF is faintly delimited and placed high on the maxilla. POB is elongated. The oribit is large and placed well behind the M3. The palate and the palatine process are large, with a circular incisor arcade. The upper teeth have a reduced number of plications in the pre- and postfossettes, whereas the protocone is asymmetrical and eloganted. The lower dentition is characterized by the U-shaped linguaflexid, with metaconid and metastylid pointed on the lingual side. The postcranial elements are usually large, although they may show different proportion related to the environment (see Boulbes and van Asperen 2019).

Equus latipes Gromova 1949 (possible synonyms *E. caballus missi, E. taubachensis*). The holotype is the right 3rd phalanx (ZIN 20664 (76)) from the Late Pleistocene Kostenki (Voronezh area, Russia), divided into *Equus latipes chosaricus* in Eastern Europe and *Equus latipes orientalis* in Eastern Siberia (Kuzmina, 1997).

Equus uralensis Kuzmina 1975 (pos syn. *E. caballus uralensis*). The holotype includes cranium parts (ZIN 29724, St. Petersburg) from the cave Black Bones in Perm region from the Late Pleistocene (Kuzmina, 1997). Overall, more than 1700 bones of *Equus uralensis* have been collected from the Urals. The skull length is 517 mm, teeth row 175.5 mm, diastema 103 mm, molar row 165-180 mm (average 176.2mm).

4.4. Africa

Representatives of the Equinae in Africa are known from the late Miocene – recent. The oldest record of hipparions is from North and East Africa (Bernor et al., 2010, 2021) at 10.5 Ma. and they extended their chronologic range in East and South Africa until sometime < 1.0 Ma. (unpublished records, Bernor personal observation). The African hipparion radiation was modest in lineage and species diversity compared to Eurasia and North America (Bernor et al., 2021). *Equus* first occurred in East Africa Omo Shungura G, 2.33 Ma. (Bernor et al., 2010).

Across Africa, Churcher and Richardson (1978) documented 191 localities of Cenozoic localities with Equidae, with concentrations in North, East and South Africa mostly of Pleistocene age. Since then, locality records have been added continent-wide. The systematics of extant *Equus* was reported by Bernor et al. (2010) and includes Grevy's zebra of East Africa (*E. grevyi*), Plains zebras of East and South Africa (*Equus quagga*, of which there are several subspecies), the mountain zebra (*Equus zebra*, of which there are 2 subspecies) and the African ass (*Equus africanus*). Bernor et al. (2021) provided a comprehensive synthesis of the Eurasian and African hipparion radiation 11.4 – 1 Ma. Recently, Van der Made et al. (2022) have presented a review of North African Plio-Pleistocene hipparions based on 19th and early 20th century descriptions. These records are mostly of isolated specimens with questionable taxonomic assignments.

Churcher and Richardson's (1978) review of African *Equus* taxonomy was extensive but most available information was restricted to living species size, coat color and pattern and osteological features of the skeleton. Long bone proportions were favored while cheek tooth size and occlusal morphology were reported (rightfully) as being highly variable for species determinations. Pleistocene Eurasian stenonine horses were advanced as being the source for the zebra-ass clade (Churcher and Richardson, 1978) and supported by recent research by Bernor et al. (2019) and Cirilli et al. (2021b) and the phylogeny of *Equus* based on Pliocene-Pleistocene North American, Eurasian and African records have supported a deep time origin from North American Pliocene aged *Equus simplicidens*, or a close relative (Cirilli et al., 2021b). Churcher and Richardson (1978) reported that *Equus caballus* was introduced into northern Africa following the invasion of Egypt by the Hyksos kings around 1580 B.C. and that introduction of *E. caballus* took place in the late 17th Century along the Cape Province by Dutch settlers.

Recently, Van der Made et al. (2022) undertook a synthesis of North African Plio-Pleistocene hipparionins recognizing several taxa from 17 regional localities which include the following taxa that they observe: *Hipparion sitifense, Hipparion (Eurygnathohippus) libycum, Hipparion (Eurygnathohippus) ambiguum* and *Hipparion (Eurygnatohippus) massoesylium.*

4.4.1 *Cremohipparion periafricanum Cremohipparion periafricanum* is a very small "dwarf" hipparionine with maxillary cheek tooth crown heights of about 50 mm and elongate and very slender mp3s.

4.4.2 *Eurygnathohippus feibeli* Bernor et al. (2020) reported a substantial assemblage of postcrania and dentitions from Sahabi and established the taxon there both on identical slenderness of the third metapodials and the existence of pli caballinids and ectostylids

on the mandibular cheek tooth dentition. Cirilli et al. (2020) reported *E. feibeli* from Tizi N'Tadderth, Morocco.

4.4.3 *Sivalhippus turkanensis.* This taxon is a relatively large late Miocene hipparionine lacking a POF, maxillary cheek teeth with lingually flattened protocone, mandibular cheek teeth with angular metaconids and metastylids and with large, robustly built mp3s and 1Ph3s.

4.4.4 *Eurygnathohippus hooijeri* Bernor and Kaiser (2006) reported a constellation of primitive and advanced features of the cranium accompanied by very tall cheek teeth (verging on a maximum crown height of 80 mm) and adult cheek teeth with weakly developed ectostylids. While the third metapodials are large (as with the cranial material), their log10 diagram is distinctly primitive comparing well with Turkish and Algerian *Cormohipparion.* The development of ectostylids on the adult mandibular cheek teeth is minimal and if further decided not to be included in the genus *Eurygnathohippus* a new generic rank would have to be considered (definitely not being *Sivalhippus, Hippotherium, Hipparion* or *Cremohipparion*).

4.4.5 *Eurygnathohippus woldegabrieli.* This taxon is intermediate in size and morphology between older *E. feibeli* and younger *E. hasumense. Eurygnathohippus woldegabrieli* was a dedicated grazer with a coarse C4 diet akin to modern zebras, wildebeests and white rhinoceroses.

4.4.6 *Eurygnathohippus afarense* The AL177-2 mandible has very large incisors that may be due to the young age of the individual and may possibly be a member of the *Eurygnathohippus hasumense* hypodigm. That being said, the mandibular symphysis of *E. afarense* (Bernor et al., 2010: Figure 35.12) is strikingly shorter than the AL425-1 mandible of *Eurygnathohippus hasumense* from the Hadar Dennen Dora member (Bernor et al., 2010: Figure 35.14) and in our opinion supports Eisenmann's (1976) original assignment.

4.4.7 *Eurygnathohippus hasumense*. *Eurygnathohippus hasumense* is a large hipparionin with elongate, narrow snout, lacking POF and with elongate-slender mp3s.

4.4.8 *Eurygnathohippus pomeli* This taxon shows a well developed, high crowned, elongate and wide ectostylids on the mandibular cheek teeth. Bernor and Harris reported that the third metapodials are more similar in their proportions to Aramis *Eu. woldegabrieli* than to Hadar *Eu. hasumense.*

4.4.9 *Eurygnathohippus cornelianus.* This taxon is recognized by its very broad mandibular symphysis, hypertrophied i1 and i2 and peg-like i3 placed distal along the midline of i2. Premaxilla shape and incisor dentitions like the mandibular symphysis. Cheek tooth crown height is high, upwards of 90 mm. maximum.

4.4.11 *Hipparion (Eurygnathohippus) libycum* The taxonomic history of this "species" is long and tortuous (Van der Made et al., 2022) with confusion whether the Lectotype specimen is a p3 or p4 or m1 or m2 (Eisenmann and Geraads, 2007). The "Lectotype" chosen by Van der Made et al. (2022; Figs. 3.1 a, 3.1b and 3.1c) is a *Eurygnathohippus* because the ?p4 has an ectostylid. Van der Made et al. (2022) state that Zouhri and Bensalmia (2005) designated AaO3647, a skull form Ahl al Oughlam as Neotype of *E. lybicus* – "*H. libycum*") whereas Eisenmann and Geraads (2007) chose the same specimen AaO3647 as their Holotype of their new species *Hipparion pomeli.* Van der Made et al. (2022: pg. 22) have set aside AaO3647 as the Neotype of *H. libycum.* According to Van der Made et al. (2022) the Lectotype of "*H. libycum*" from Carriere Brunie has a caballoid double knot and ectostylid (their Fig. 7.1). Eisenmann and Geraads (2007) in naming the species *"Hipparion"* (= *Eurygnathohippus* of Bernor et al., 2010 and 2021) *pomeli* from Ahl al Oughlam dismissed *"Hipparion libycum"* as being an invalid taxon due to insufficient skeletal material for confident identification. Suffice it to say, the cheek teeth figured by Van der Made et al. (2022) for their *extended* definition of *"Hipparion (E.) libycum"* differ in no essential details from East African hipparions of the *Eurygnathohippus woldegabrieli – E. hasumense – E afarense* lineage and do not constitute a clearly recognizeable species as required by Article 75.5 of the Code. The extension of the nomen *E. libycum* by Van der Made et al. (2022) to

material from other localities has no real scientific value. There are no absolute dates, either radioisotopic nor magnetostratigraphic cited for *E. libycum* by Van der Made et al. (2022), but occurrences cited by them lack *Equus* and the resulting presumption is the *E. "libycum"* is of Pliocene age, and with the absence of *Equus* > than 2.6 Ma.

4.4.12 *Hipparion (Eurygnathohippus) ambiguum* Given that it is a P2 it, in itself, is not sufficient to recognize a species of hipparionin horse. Also, not being an adult mandibular cheek tooth with an ectostylid, it cannot be certainly be identified as being a species of *Eurygnathohippus.* As such, *"H. ambiguum"* is appropriately named being an ambiguous taxon. Hopwood (1937) considered *"Hipparion ambiguum"* as being a synonym of *"Hipparion libycum".* As with other North African hipparions, *"Hipparion ambiguum"* has a long, tortuous taxonomic history as cited by Van der Made et al. (2022: Thomas, 1884; Pomel, 1897; Hopwood, 1937; Arambourg, 1970; Eisenmann and Geraads, 2007). Van der Made et al.'s (2022, pg. 55) citation of hipparions from Hadar, Koobi Fora, Olduvai Beds 1 and 2, Ahl al Oughlam, Ain Boucherit and Oued Aklrech and Ain Jourdal being similar …"in development of ectostylids and moderately gracile metapodials" has no realistic systematic value when one realizes that the Type of *"H. ambiguum"* is a poorly documented P2.

4.4.13 *Hipparion (Eurygnatohippus) massoesylium* These teeth in themselves do not constitute referral to *Eurygnathohippus* because they are not adult mandibular cheek teeth with ectostylids. Hopwood (1937) considered *"Hipparion" massoesylium* to be a synonym of *"H". libycum.* Van der Made et al. (2022) referred additional material from Ain Hanech (Figs. 7.5, 7.11) and Lake Ichkeul (Figs. 7.7-7.10) which included lower cheek teeth in order to assert affiliation with *Eurygnathohippus.* Hopwood (1937) considered *"Hipparion" massoesylium* to be a synonym of *"H". libycum*; an opinion followed by most later authors (Van der Made et al., 2022). Van der Made et al. (2022) have attempted to extend the nomen *"H. (E.) massoesylium"* to specimens from Puits Karoubi, Ain Hanech, Olduvai, the Upper Ndolanya Beds, the KBS Mb, Omo Shungura members F, G/K and H because of their small size. In itself, the small size of the type material of *Eurygnathohippus "massoesylium"* is insufficient for species recognition because there are other small hipparion species that occur in Eurasia and Africa from which it cannot be certainly distinguished by the Holotype maxillary cheek teeth. Van der Made et al. (2022) have estimated the age of Ain Hanech as being 1.7 Ma based on magnetostratigraphy, ESR dating, biochronology and sedimentation rates.

4.4.14. *"Hipparion" sitifense "Hipparion sitifense"* can not be substantiated as a legitimate species because the original type series is insufficient to distinguish it from other taxa such as *Cremohipparion periafricanum, Cremohipparion nikosi* even *Eurygnathohippus feibeli* (for which a lower cheek tooth is needed). Previous referrals by Bernor et al. (1987) to *"Hipparion"* cf. ?*sitifense* have been corrected by Bernor et al. (2020) and Cirilli et al. (2020) to the presence at Sahabi, Libya and Tizi N'Taddehrt, Morocco of a small species *Eurygnathohippus feibeli* and a dwarf species *Cremohipparion* sp., apparently similar to *Cremohipparion periafricanum* and *Cremohipparion nikosi* (possible junior synonym of *C. periafricanum* under study by Bernor and Cirilli). The age of Saint Arnaud cemetery is believed to be Pliocene age (MN14?), but this is far from being certain. The extended application of the nomen *"Hipparion sitifense"* to other North African "small hipparions" by Van der Made et al. (2022) is inappropriate and more than likely confuses *"H. sitifense"* with small *Cremohipparion* and *Eurygnathohippus* species.

4.4.15. *Equus numidicus* The upper cheek teeth show a morphology similar to *E. stenonis*, with a short protocone (which becomes longer serially from M1 to M3); pree and postefossettes well developed with large mesostyles. The lower cheek teeth have the typical stenonine V-shaped linguaflexid separating metaconid metastylid, with the metastylid pointed lingually. The metapodials are elongated, with large and robust proximal, medial and distal widths.

4.4.16 *Equus tabeti* No complete crania are known from this species, although two small palates are preserved. The upper cheek tooth rows have small dimenstion, and the teeth shows a reduced protocone, a large and squared mesostyle and a round hypocone. The lower cheek teeth have the V-shaped linguaflexid, with the metastylid pointed on the lingual side. The ectoflexid is reduced in the premolars and deeper in the molars. Postcranial elements have a small dimension if compared with the other Early Pleistocene species from North Africa, and have a very slender morphology. This is the most gracile species of the Early Pleistocene in North Africa.

4.4.17 *Equus koobiforensis* The skull is large with an undulated forehead profile. In lateral view the skull has a moderate length snout, nasal notch is retracted to mesostyle of P3 and the POF is very reduced and placed high on the maxilla. The facial-maxillary crest is modestly developed and the orbit is placed well behind the M3. In ventral view the right half of the snout is broken away with only left I2 and I3 in place with open infundibula. The maxillary cheek tooth dentition has P2 with a short anterostyle and short ovate protocone not connected to protoloph; P3 is triangular shape with a slight connection to protoloph and protocone is likewise triangular shaped with no connection to the protoloph; P2 has a labially squared mesostyle, while P3 and P4 have mesiodistally constricted and labially pointed mesostyles; all premolars have weakly developed fossette ornamentation; P2 has a moderately deeply incised hypoglyph and P3 and P4 have deeply incised hypoglyphs; M1–M3 have elongate, lingually flattened protocones with only M1 having a clear connection to the protoloph; all molars have labially pointed mesostyles and weakly developed fossette ornamentation (edited from Bernor et al. 2019).

4.4.18 *Equus oldowayensis* Churcher and Hooijer, 1980 identified *E. oldowayensis* as being a large horse overlapping in size with *Equus grevyi.* Cooke (1963, table 4) assigned a large equid from the Omo deposits to *E. oldowayensis* Hopwood 1937, "or possibly *E.* aff. *grevyi*", without discussion (Bernor et al., 2010). Hooijer (1976) reported a large *Equus* from the Omo and Churcher and Richardson (1978) recognized *Equus* (*Dolichohippus* [= Grevy's zebra line]) from Omo Shungura Members F through J. In order to stabilize the taxon *Equus oldowayensis* Churcher and Hooijer (1980) selected a mandible, BMNH M14184 as the Neotype of *E. oldowayensis.* Churcher and Hooijer (1980) reported on the extensive Olduvai *Equus* material in the Rijksmuseum van Natuurlijke Histoire, Leiden. *Equus oldowayensis* was a large horse with a broad muzzle and horizontal incisive row and likely adapted to short grass feeding. Olorgesailie has a complete skull, lacking the occipital region collected from "Hyaena Hill", 0.99 Ma. This specimen is currently under study by Bernor and Potts (in progress) and would appear to conform with the concept of Churcher and Hooijer (1980). This specimen has been referred to briefly by Bernor et al. (2019).

Churcher and Hooijer, 1980 reported a large horse overlapping in size with *Equus grevyi*, with a large, broad, incisor arcade; infundibula present on I1 and I2, possibly absent on I3, P2 and P3 protocones shorter and rounder than on P4 and persistently longer on molars; protocone strongly linked to protoloph; pli caballins vestigial or absent; fossette plications simple. Mandibular incisors as in maxillary incisors having distinct infundibula; lower cheek teeth have vestigial metaconid on p2 and rounded or slightly elongate on p3-m3; metastylids are rounded to square shaped; linguaflexids very shallow on p2 and V-shaped on p3-m3. Third metapodial variable in length and slenderness (edited from Bernor et al. 2019).

4.4.19 *Equus capensis* Eisenmann (2000) reported a well preserved skull from Elandsfontein (South Africa), quite large but not like a Grevyi' zebra skulls. In this skull, the distance between the palate and the vomer is relatively short, and the muzzle is wide. The analyses provided by Eisenmann (2000) reported similarities with the Cape quaggas. Postcranial elements suggest that it was a large equid, larger than *E. zebra* and *E. quagga* (Eisenmann 2000). Kaiser and Franz-Odendaal (2004) interpreted *E. capensis* paleodiet to indicate a mixed feeding niche. The relationship between East African *E. oldowayensis* and S. African *E. capensis* is uncertain.

4.4.20 *Equus mauritanicus* The skulls from Tighenif are medium sized, with an undulated forehead profile. The muzzle is short, and the nasal notch extend above the P2. The maxillary crest is developed up to the P4/M1 boarder, whereas the POF is almost oblitered. The orbit is placed above the posterior border of the M3, and the braincase is deflected and relatively small. The upper dentirion has short protocones, which become serially longer in the molars, a short and squared mesostyle and a round hypocone. The lower dentiton has the typical V-shaped linguaflexid, with the metastylid pointed on the lingual side. The ectoflexid is deep, both in premolars and in the molars. Third metapodials are short and stout, reduced than *E. numidicus* and *E. oldowayensis*.

4.4.21 *Equus melkiensi*s Bagtache et al. (1984) nominated two paratypes: a left m2, I.P.H. Allo. 61-1969 and a left metatarsal III, I.P.H. Allo. 61-1834. The left m2 is stated to be "typical stenonine that Bernor et al. (2010) reconstructed from photographs having elongate-rounded metaconid and rounded-square metastylid, V-shaped linguaflexid, short preflexid with a strong mesiolingual pli; postflexid small and labiolingually constricted, shallow ectoflexid not invading between pre- and postflexid

4.4.22 *Equus algericus* There is a substantial suite of third metapodials in the type assemblage which are remarkable for their stocky build.

4.4.23 *Equus grevyi* It is a large-headed and long-legged horse with extremely large ears. Along with *E. grevyi*, Churcher and Richardson (1978) referred *E. numidicus, E. oldowayensis* and *E. capensis* to the subgenus *Dolichohippus.* The skull is very elongated, with an undulated linear forehead profile. The muzzle is long and slender, and the nasal notch extend above the P2 mestostyle. The maxillary crest is developed up to the P4/M1 boarder, whereas the POF is faintly delimited and placed high on the maxilla. The infraorbital foramen is located high above the P4 mesostyle. The orbit is placed well behind the posterior boreder of the M3, and the braincase is deflected and elongated. The upper dentition shows an elongated protocone, longer in the molars than premolars, a large and squared mesostyle and a round hypocone. The lower dentiton has a V-shaped linguaflexid, with the metastylid pointed on the lingual side. The ectoflexid is more developed in molars than premolars. Third metapodials are elongated and slender.

4.4.24 *Equus quagga* The skull is medium sized, with an sinuous lateral profile. The muzzle is short, and the nasal notch is extended in front of the P2. The maxillary crest is developed up to the P4/M1 boarder, whereas the POF is faintly delimited and placed high on the maxilla. The infraorbital foramen is placed above the P4 mesostyle. The orbit is placed above the posterior boreder of the M3, and the braincase is deflected and relatively small. The upper dentition has short protocones, more elongated in molars than premolars, a small and squared mesostyle and a narrow hypocone. The lower dentiton has a V-shaped linguaflexid, with the metastylid pointed on the lingual side. The ectoflexid is well developed, more elongated in the molars than premolars. Third metapodials are short but roboust.

4.4.25 *Equus zebra* Groves and Ryder (2000) consider the mountain zebras to be distinct species, whereas Moodley and Harley (2005) and Moodley et al. (2006) support their classification into two subspecies. The skull is elongated, with an ondulated lateral profile. The muzzle is long and slender, and the nasal notch extend above the P2 mestostyle. The maxillary crest is developed up to the P4/M1 boarder, whereas the POF is reduced and placed high on the maxilla. The infraorbital foramen is located high above the P4 mesostyle. The orbit is placed above the posterior boreder of the M3, and the braincase is deflected and elongated. The upper dentition shows an elongated and asymmetrical protocone, longer in the molars than premolars, a small and squared mesostyle and a round hypocone. The lower dentiton has a V-shaped linguaflexid, with the metastylid pointed on the lingual side. The ectoflexid is more developed in molars than premolars. Third metapodials intermediate in size between *E. grevyi* and *E. quagga*.

4.4.26 *Equus africanus* The African ass has a withers height ranging from about 1.15 to 1.25 cm. The skull is medium sized, with an almost linear lateral profile. The muzzle is short,

and the nasal notch extends above the P2 mesostyle. The maxillary crest is developed up to the P4/M1, whereas the POF is faintly delimited and placed high on the maxilla. The infraorbital foramen is placed high above the P4 mesostyle. The orbit is located behind the posterior border of the M3, and the braincase is deflected and relatively small. The upper dentition has short protocone with a relevant intoflection of the lingual side, a small and squared mesostyle and a narrow hypocone. The lower dentiton has a V-shaped linguaflexid, with the metastylid pointed on the lingual side. The ectoflexid is not well developed.

6. Paleoecology

6.1. _Paleobotany and Paleoenvironments_

Global climate experienced a general cooling during the Neogene with a progressive aridification being recorded from the Late Miocene onwards in the Northern Hemisphere which led to the development of a marked north–south temperature gradient similar to present–day conditions (Barnosky, 1987; Eronen et al., 2012). Arid conditions developed first in North America and Eastern Eurasia, and later in Europe which retained more humid conditions until the Pliocene (Eronen et al., 2012). Around 5 Ma Europe and the North Mediterranean region in particular, was characterized by a warm and humid climate with average temperatures that reached their peak (up to 4 °C higher than present–day) between 4.4 and 4 Ma during the Pliocene Climate Optimum (PCO) (Fedorov et al., 2013). Tropical and subtropical vegetation dominated the landscape until the Late Pliocene when a progressive deterioration of the climatic conditions and the establishment of the typical Mediterranean precipitation regime (dry summers) around 3.4 Ma (Suc, 1984) led to the gradual disappearance of thermophilous plants that require high levels of humidity all year round (e.g., Taxodiaceae) and the spread of herbaceous taxa (notably Artemisia) (Michaux et al., 1979; Bertini, 2010). During the Pliocene/Pleistocene transition the onset of the Quaternary Ice Age resulted in a decrease of mean temperatures in the Northern Hemisphere determining an alternation of cool temperate glacials and warm humid interglacials dominated by a low–amplitude 41 ka periodicity (Head and Gibbard, 2015). In this phase xeric savannah–like environments developed during glacials while subtropical forests persisted during interglacial phases (Bertini, 2010). The increase in length and intensity of glacial cycles following the transition to a 100 ka periodicity (Early–Middle Pleistocene Transition event) (Head and Gibbard, 2015), further exacerbated climatic conditions leading to the disappearance of all subtropical ecosystems in the region which were replaced by temperate deciduous forests and spreading Mediterranean vegetation during Middle Pleistocene interglacials (Suc and Popescu, 2005; Bertini, 2010; Combourieu–Nebout et al., 2015). Through the Late Pleistocene the increased duration of cold phases which reached their last peak during the Last Glacial Maximum (~20 ka) (Mix, 2001), resulted in the development of steppe and tundra–like environments, with Mediterranean vegetation being restricted to lower latitudes during glacials (Bertini, 2010). Interglacials were still dominated by deciduous forests, but plants adapted to low temperatures (e.g., *Fagus*) were more frequent at higher altitudes and latitudes (Combourieu–Nebout et al., 2015).

6.2. _Overview of North American fossil taxa from Pliocene and Pleistocene with dietary assignments_

The major evolution and diversification of equids occurred in North America despite the fact that a number of successive dispersals took place to the Eurasia. Equidae apparently evolved in isolation from Eurasia in North America from the middle Eocene to the late Oligocene (MacFadden, 1992). During the Tertiary, equids were very widespread in North America. In fact, at most fossil localities, they are the most common medium- to large-sized mammals recovered (MacFadden, 1992). Equids achieved their

maximum diversity in the late Miocene (MacFadden, 1992; Hulbert, 1993) resulting in the evolution of the subfamily Equinae (Hulbert and MacFadden, 1991).

The Miocene was also a time of craniodental reorganization of the equid skull (Radinsky, 1983, 1984). These trends began in parahippine and merychippine equids and eventually led to the genus *Equus*, which is thought to have evolved from *Dinohippus* in the Pliocene (MacFadden, 1984; Hulbert, 1989). The dramatic changes in equid skulls, teeth and limbs have long been thought to reflect evolutionary adaptations to a changing environment – often thought of as evidence that grasslands expanded during this time (e.g., Osborn, 1910; Scott, 1937; Simpson, 1944; Stirton, 1947; Webb, 1977, 1983; Janis, 1984, 1993; Webb and Opdyke, 1995). Thus, once horses became hypsodont, it has traditionally been assumed that they subsisted on grasses. Because the fossil record of horses is rich, it offers an excellent opportunity to test such dietary assumptions and to track paleoenvironmental and vegetational trends through time via such dietary proxies as hypsodonty indices, mesowear and microwear.

North American fossil equid taxa spanning from the Late Miocene (Clarendonian) through the Pleistocene (Rancholabrean) were studied by analyzing microwear, mesowear and hypsodonty (Supplementary Table 5). Data from North American fossil derived Equinae ranging from the late Miocene (Clarendonian) through the Pleistocene (Rancholabrean) were obtained from Semprebon et al., 2016 (microwear; N = 349), Mihlbachler et al., 2011 (mesowear; N = 6498), and Semprebon et al., 2019 (hypsodonty indices; N=62). These data were compared to published microwear data on extant ungulates (Solounias and Semprebon, 2002) (N=809/50 species) to reconstruct the dietary paleoecology of derived Equinae through time.

Microwear data was obtained by a single, trained observer (GS) using a stereomicroscope (35× magnification) and following the analytical technique of Semprebon (2002), Solounias and Semprebon (2002) and Semprebon et al. (2004). This involved quantifying the average number of pits (rounded features) vs. average number of scratches (elongated features) per taxon within a 0.16 mm^2 area using an ocular reticle to distinguish between browsers and grazers. These results were compared to an extensive extant ungulate database (Semprebon, 2002; Solounias and Semprebon, 2002).

Large pits and gouges were scored as present or absent – the latter scored as present if more than four large pits per microscope field were observed and the latter as present or absent within the 0.16 mm^2 area. After this, the percentage of individuals within each taxon with these variables was calculated. The texture of scratches was scored qualitatively as being mainly fine, mainly coarse, or a mixture of fine and coarse textural types following protocols for discriminating these textural differences described in Solounias and Semprebon (2002) and Semprebon et al. (2004). A scratch width score (SWS) was obtained by giving a score of 0 to molars with mostly fine scratches, 1 to molars with a mix of fine and coarse textures, and 2 to those with mostly coarse scratches. An average scratch width score was calculated by averaging the individual sws scores for each taxon. The percentage of raw scratches per taxon falling into a low raw scratch range of 0–17 scratches was used to distinguish mixed feeders that alternate between browsing and grazing (after Semprebon, 2002 and Semprebon and Rivals, 2007).

Mesowear, which measures both attritional (tooth-on-tooth) and abrasional (food-on-tooth) tooth wear, was scored by observing molar cusps macroscopically in buccal view and determining cusp sharpness (i.e., sharp, round, blunt) and the degree of occlusal relief (high or low) (Fortelius and Solounias, 2000). Because occlusal relief and cusp shape are not actually independent variables, mesowear was treated as a single variable such that cusp apices were assigned to one of 7 stages along a continuum that ranged from sharp cusps with high relief to blunt cusps with low relief. This was done by comparing fossil molars to a mesowear "ruler" (Mihlbachler et al., 2011) which employs seven extant *Equus* tooth cusps that range from sharp cusps with high relief (stage 0) to blunt cusps with no relief (stage 6). If cusp tips were concave upwards (i.e., had negative relief), they

were assigned to stage 7. An average mesowear value from each fossil tooth sample was calculated to derive a mesowear score (mws) for that taxon.

Hypsodonty data was provided by Christine Janis (Semprebon et al., 2019). Hypsodonty was measured as the crown distance from the base of the crown to the tip of the protoconid (crown height) divided by the labio-lingual width of the third, lower and unworn molar (providing the crown height or m3 ratio). The width of the molar was measured between the protoconid and the entoconid (occlusal surface).

SAMPLE #	TAXON	N	LOCALITY	AGE
			**LATE MIOCENE**	
1	*Pseudhipparion hessei*	28	Ogallala Fm., Donley Co., Texas	Clarendonian
2	*Calippus martini*	6	Ash Hollow Fm., Merritt Dam Mbr., Cherry Co., Nebraska	Late Clarendonian
3	*Cormohipparian occidentale*	17	Ash Hollow Fm., Xmas Quarry Zone, Merritt Dam Mbr., Cherry Co., Nebraska	Late Clarendonian
4	*Hipparion tehonense*	12	Clarendon Beds, MacAdams Quarry, Donley Co., Texas	Late Clarendonian
5	*Dinohippus leidyanus*	7	Snake Creek Fm., Johnson Mbr., Sioux Co., Nebraska	Late Hemphillian
6	*Dinohippus* sp.	6	Ogallala Group, Edson Area, Sherman Co., Kansas	Late Hemphillian
7	*Dinohippus interpolatus*	12	Eden Fm., Riverside Co., California	Late Hemphillian
8	*Dinohippus leidyanus*	16	Redington Area, Old Cabin Quarry, Pima Co., Arizona	Late Hemphillian
9	*Dinohippus* sp.	17	Quiburis Fm., Redington Area, Old Cabin Quarry, Pima County, Arizona	Late Hemphillian
			**PLIOCENE**	
10	*Nannipus aztecus*	21	Bone Valley Fm., Various Quarries, Florida	Latest Hemphillian
11	*Pseudhipparion simpsoni*	12	Bone Valley Fm., Polk Co., Florida	Latest Hemphillian
12	*Neohipparion eurystyle*	10	Bone Valley Fm., Polk and Hardee Cos., Florida	Latest Hemphillian
13	*Cormohipparion emsliei*	9	Bone Valley Fm., Various Localities, Florida	Latest Hemphillian
14	*Equus simplicidens*	6	Glenns Ferry Fm., Hagerman Horse Quarry, Twin Falls Co., Idaho	Early Blancan
15	*Equus simplicidens*	16	Kiem Fm., Sand Draw Local Fauna, Ainsworth Area, Brown Co., Nebraska	Early Blancan
16	*Equus* sp.	8	Dry and Northern Whitlock Mtn Localities, Graham Co., Arizona	Early Blancan
17	*Equus* sp.	18	St. David Fm., Benson Area, Cochise Co. Arizona	Early Blancan
18	*Nannipus peninsulatus*	9	Ogallala Grp., Crosby Co., Texas	Blancan
			**PLEISTOCENE**	
19	*Equus* sp.	11	Lake Agassiz, North Dakota	Late Blancan
20	*Equus* sp. A	23	Bermont Fm., Leisey Shell Pit 1A, Hillsborough Co.,	Early Irvingtonian

Table S7. North American fossil equid taxa spanning from the Late Miocene (Clarendonian) through the Pleistocene (Rancholabrean).

Figure S2 shows equid hypsodonty and mesowear trends through time from the Miocene to the Recent. In the middle Miocene, the “Derived Equinae” undergo a consistent increase in hypsodonty (which is consistent with some closed- and open-habitat mixed feeders) and continue this trend through the Pliocene and Pleistocene and into the Recent - attaining hypsodonty levels consistent with extant open-habitat mixed feeders and regular grazers. A similar trend is seen with mesowear although the trend is a bit less consistent.

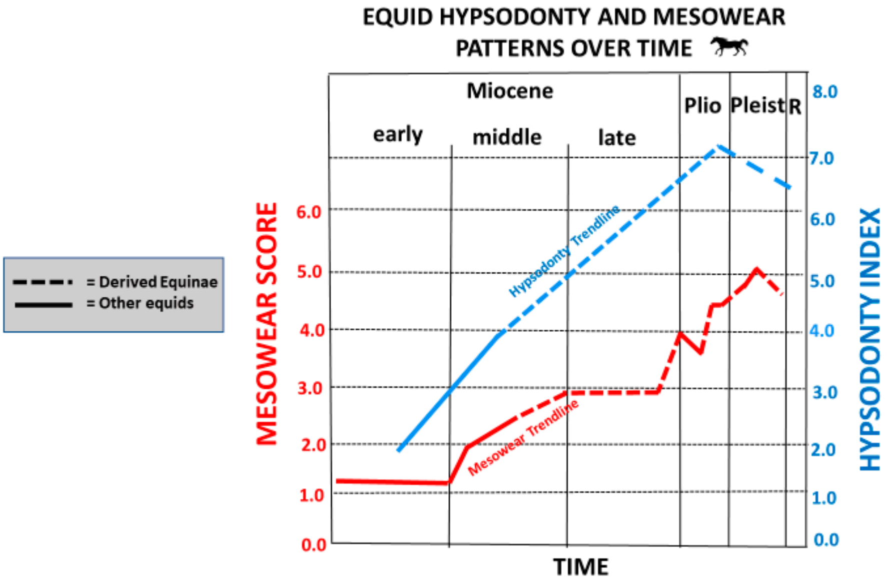

Figure S2. Equid hypsodonty and mesowear trends from the Miocene to the recent.

Figure S3 represents a synthesis of scratch width score trends (microwear), mesowear trend (i.e., MWS trendline), low scratch range scores (i.e., SWS trendline) from microwear (% scratches per taxon that fall between 0–17) and dietary assignments obtained via the low scratch range calculation for Derived Equinae. Dietary assignments based on microwear of equid taxa are represented by symbols used to represent the mesowear scores (diamonds = leaf- dominated browsers, circles = regional or seasonal mixed feeders, squares = grazers). A mesowear trend toward more abrasion in the Equidae diet beginning in the Miocene is consistent with microwear results in that, it was in the early Miocene scratch textures (sws) shifted toward toward coarser scratch textures (i.e., higher sws scores).

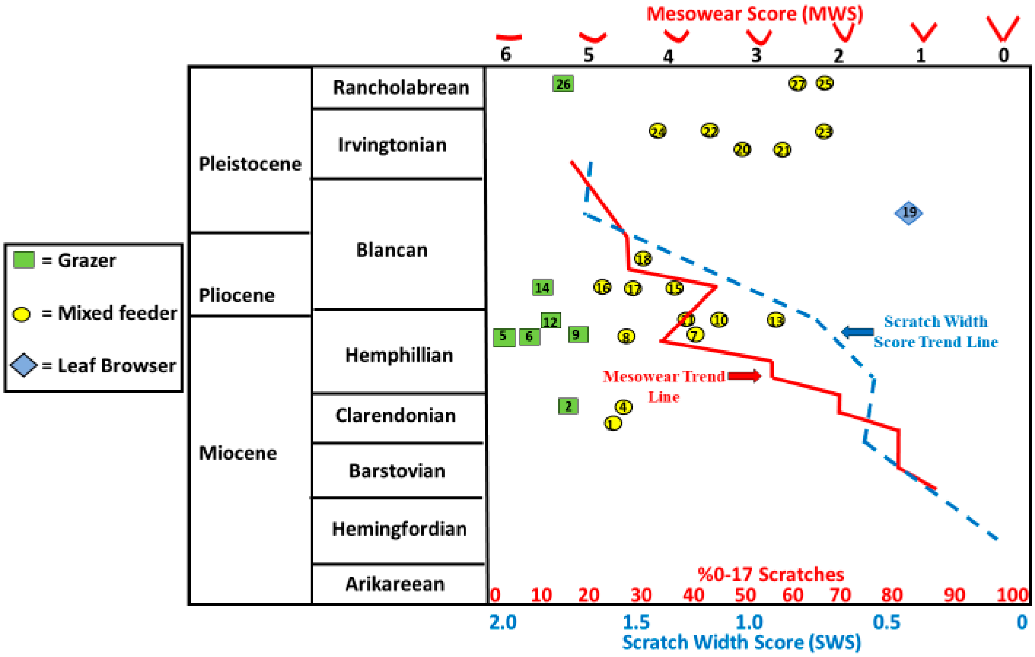

Figure S3. Synthesis of scratch width score trends (microwear), mesowear trend (i.e., MWS trendline), low scratch range scores (i.e., SWS trendline) from microwear (% scratches per taxon that fall between 0–17) and dietary assignments obtained via the low scratch range calculation for Derived Equinae.

In the middle Miocene, equid mesowear (Figure S4) continued the trend toward greater abrasion. Microwear results (0-17 low scratch range scores) show that this trend toward greater abrasion revealed by mesowear was due to the Derived Equinae engaging mostly on high-abrasion grass consumption. SWS trends mirror that of the mesowear score trends through time. Scratch width is a measure of relative abrasion in microwear whereas, mesowear scores are a measure of relative abrasion in gross cusp tip shape.

In the late Miocene, the Derived Equinae continued with coarse microwear but were both grazing and mixed feeding (Figure S4). Scratch textures are coarser than those seen in the middle Miocene indicating a shift in habitat toward more open and drier environments in the late versus the middle Miocene.

The shift toward a coarser type of feeding in Derived Equinae continued into the Pliocene (Figure S4). Scratch textures are coarser overall, which matches the increased abrasion seen in mesowear at this time.

Interestingly, in the Pleistocene, only one species in the genus *Equus* studied here (*Equus* sp. from Fairbanks Alaska) has microwear consistent with grazing. The other taxa alternated between coarse grass and browse (i.e., were mixed feeders).

Figure S5 shows typical microwear results for extant ungulates of known and particular dietary behavioral categories.

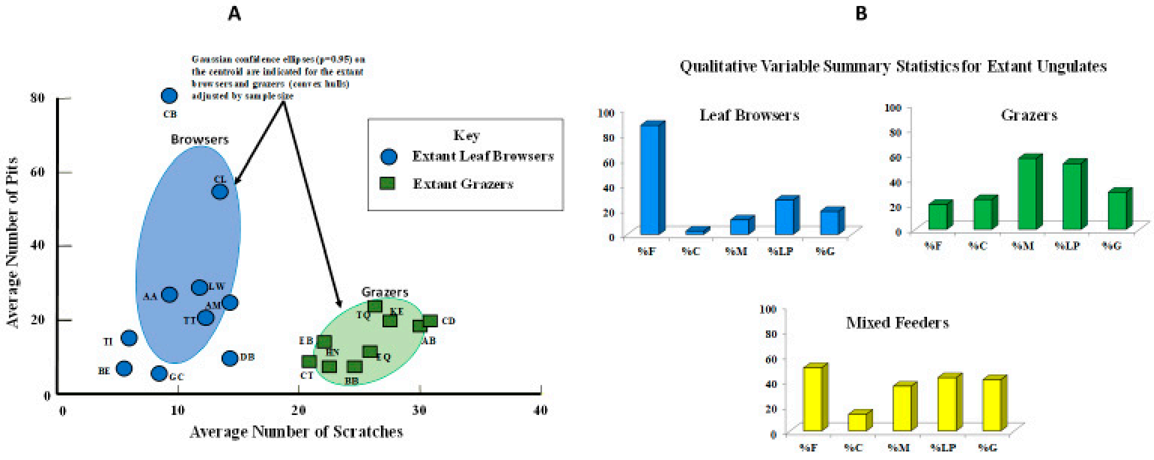

Supplementary Figure S5. Microwear results and graphical representation for extant ungulates.

Figure S6A-C demonstrates the usefulness of a bivariate plot of the average number of scratches versus the average number of pits per taxon for extant leaf and fruit browsing and grazing ungulates as it is clear that there is no overlap in microwear results between the extant grazing and browsing taxa (the latter have fewer scratches). Figure S7B shows bar charts that represent the percentages of individuals per extant ungulate taxon that possesses predominantly finely textured scratches (F), coarsely textured scratches (C), a mixture of finely and coarsely textured scratches (M), more than 4 large pits (LP) and Gouges (G) in the microscope counting area (data from Semprebon, 2002 and Solounias and Semprebon, 2002). It can be seen in Figure S7B that typical extant browsing ungulates possess mostly finely textured scratches but grazers and mixed feeders have fewer finely textured scratches (more coarsely textured scratches).

Figure S6A shows average scratch and pit quantitative results for derived Equinae from the late Miocene, Pliocene, and Pleistocene plotted in reference to Gaussian confidence ellipses (p = 0.95) on the centroid for the extant browser (B) and grazer (G) data

adjusted by sample size from Semprebon (2002) and Solounias and Semprebon (2002). Bar charts for the qualitative microwear variables are also shown in Figure S6A.

Qualitative microwear results for late Miocene Derived Equinae taxa (Figure S6A bar charts) show higher levels of gouging, large pits, and coarser scratch textures than are typical of extant browsing, mixed feeding and grazing ungulates.

Average scratch and pit and qualitative results for the Pliocene derived Equinae are shown in Figure S6B. Note that most of the taxa actually fall in the gap between extant leaf browsing and grazing ecospace confidence limits (and thus were most likely mixed feeders based on their low scratch ranges) while others have low scratch range results consistent with extant grazers but fall above the grazing morphospace because of relatively high pit counts. Qualitative microwear results for Pliocene taxa (bar charts) show that scratch textures got coarser in the Pliocene versus the late Miocene equids even though gouging and large pitting are pretty similar between these time periods.

Average scratch and pit and qualitative results for the Pleistocene derived Equinae are shown in Figure S7C. Note that some members of the Genus *Equus* fall in the extant leaf browsing ecospace, some within the extant grazing ecospace, and most in between the leaf-browsing and grazing ecospaces. The bar charts in Figure S7C show that Pleistocene derived Equinae tend to have large pitting, gouging and scratch textures similar to extant grazers or mixed feeders but like Pleistocene forms, more coarse scratch textures than late Miocene forms. An indicator of relative abrasion is scratch width (Semprebon et al., 2016).

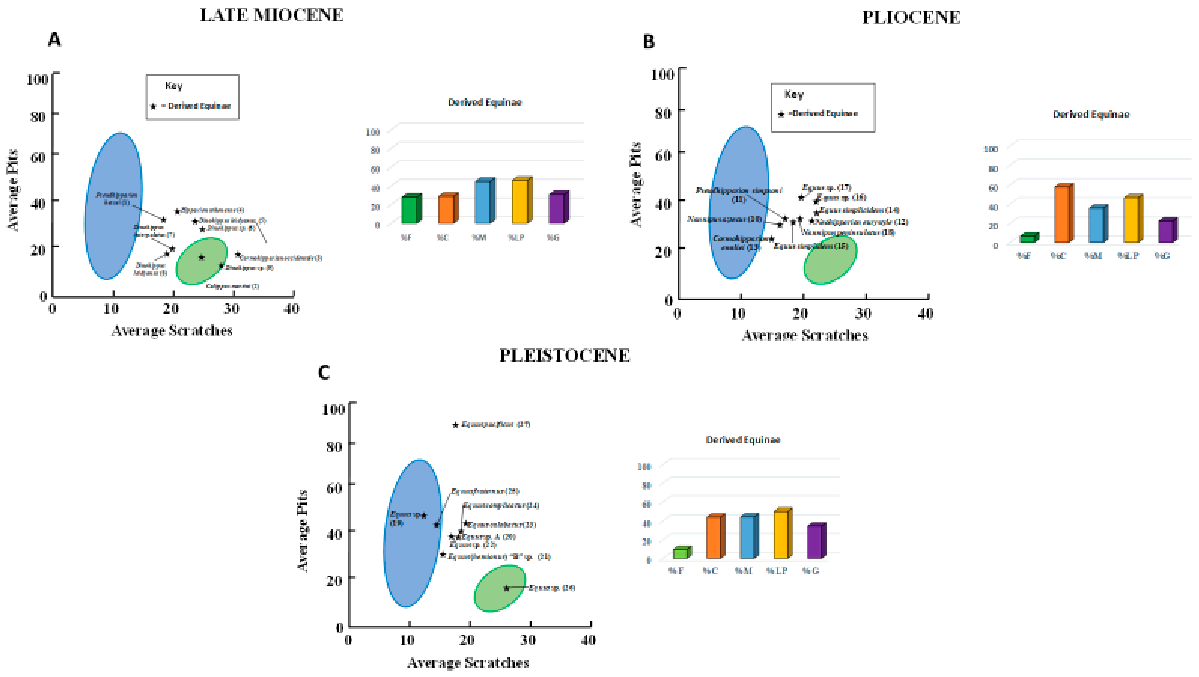

Figure S6. Bivariate plot of the average number of scratches versus the average number of pits per taxon for extant leaf and fruit browsing and grazing.

Figure S7 depicts scratch width score (sws) temporal trends for derived Equinae (blue) versus more primitive horses (red) from the Miocene through the Recent compared to the mean sws for extant leaf browsers and grazers. While less derived equids had scratch width scores that declined steadily throughout the Miocene, derived Equinae show a trend toward very high scratch width scores from the Miocene through the Recent. This rise in abrasion parallels that seen in mesowear (Figure S4) and hypsodonty levels (Figure S3)

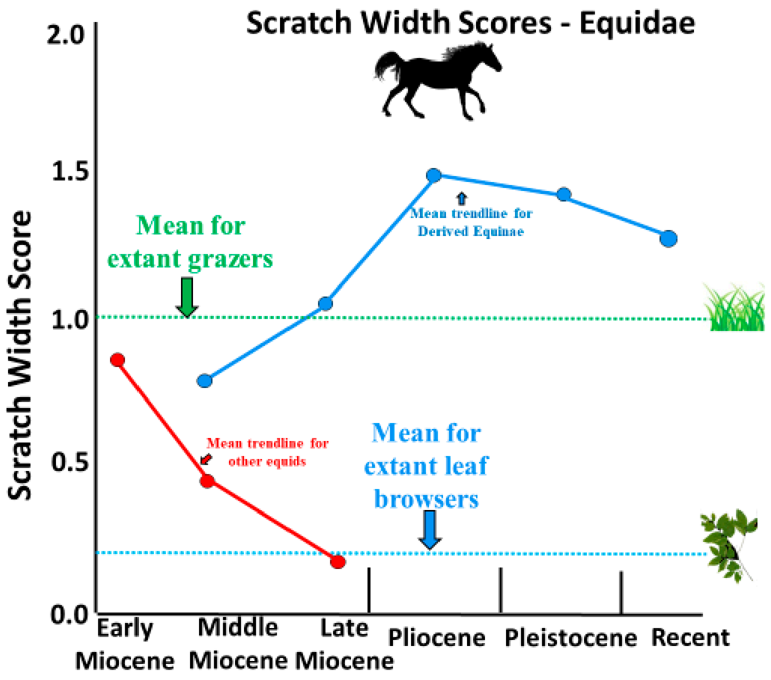

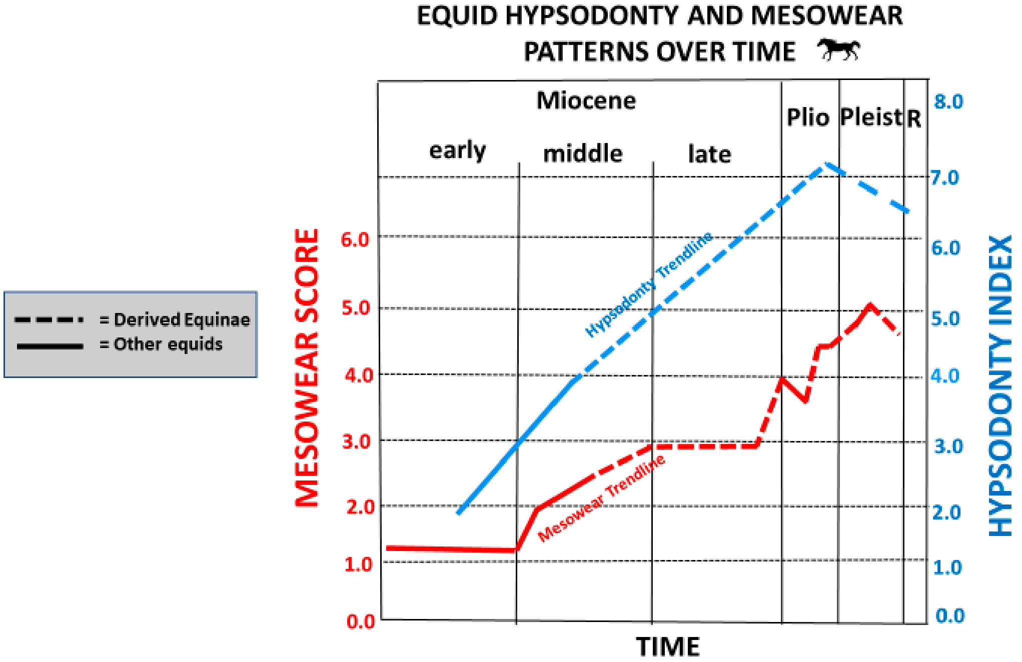

Figure S7. Scratch width score (sws) temporal trends for derived Equinae (blue) versus more primitive horses (red) from the Miocene through the recent compared to the mean sws for extant leaf browsers and grazers.

7. Climate and Evolution

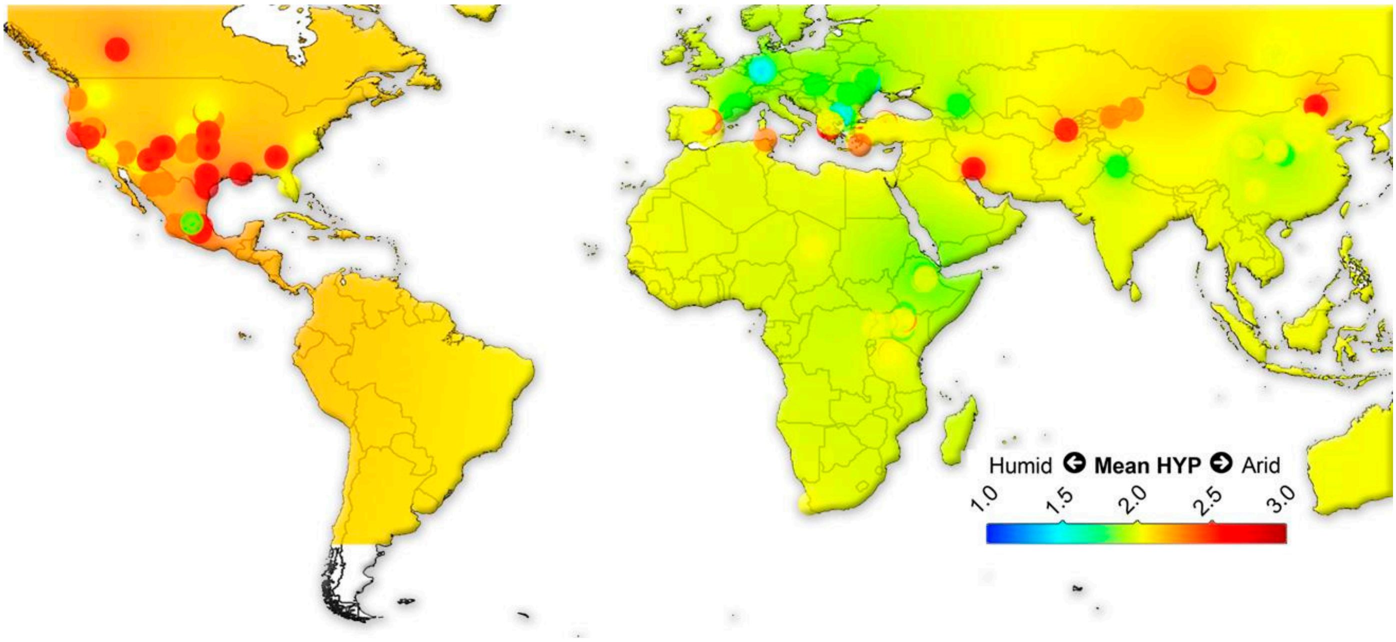

Figure S8. Spatial distribution of the large herbivorous genera mean ordinated crown height through time ranges 7 to 4 Ma in North, Central and South America, Eurasia and Africa. The mean ordinated hypsodonty map represents the paleoclimatological conditions grading from most humid (blue) to most arid (red), without the coded number given in Table S4. The mean ordinated hypsodonty values are represented by the color-coded circles indicate the spatial position of the localities that mean hypsodonty scores calculated (Table S5). IDW interpolation algorithm hypothetically interpolates no data (no locality) area based on the actual data. These areas should be ignored.

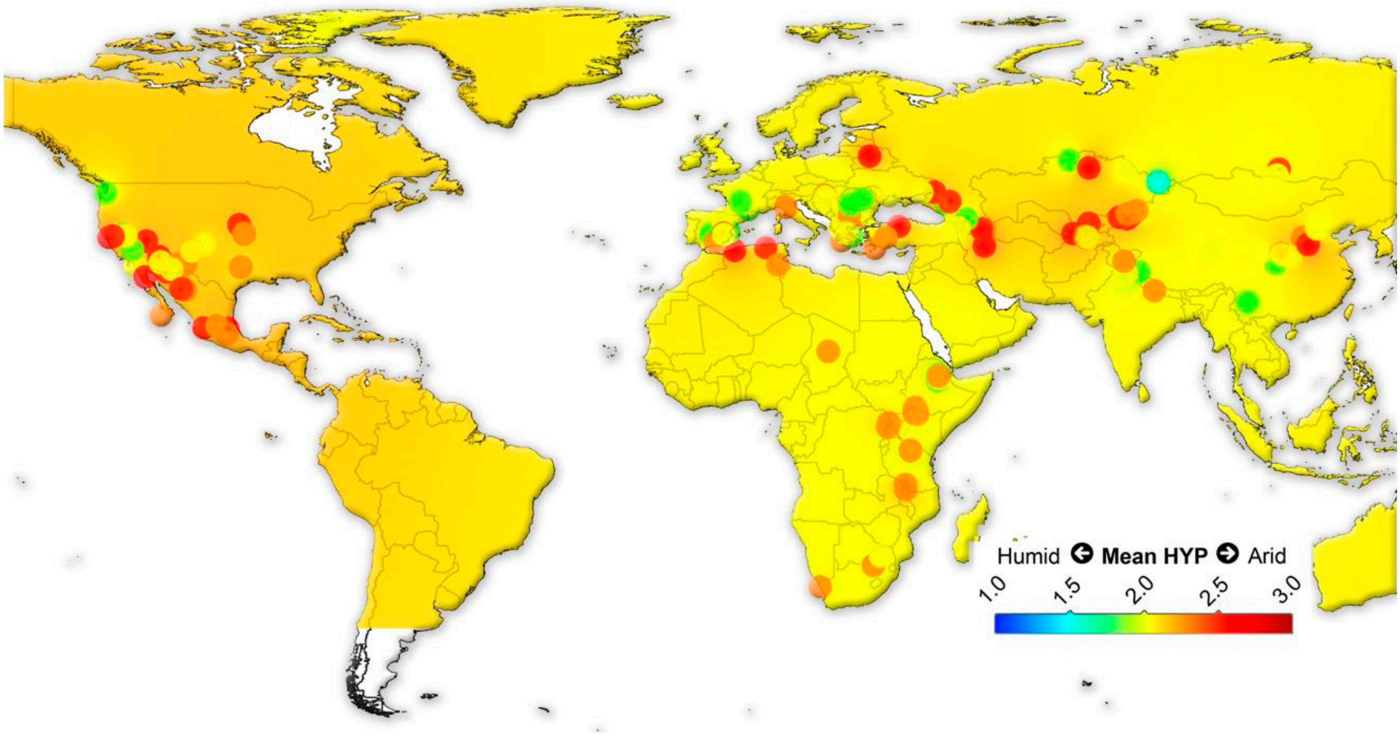

Figure S9. Spatial distribution of the large herbivorous genera mean ordinated crown height through time ranges 4 to 2.6 Ma in North, Central and South America, Eurasia and Africa. The mean

ordinated hypsodonty map represents the paleoclimatological conditions grading from most humid (blue) to most arid (red), without the coded number given in Table S4.The mean ordinated hypsodonty values are represented by the color-coded circles indicate the spatial position of the localities that mean hypsodonty scores calculated (Table S5). IDW interpolation algorithm hypothetically interpolate no data (no locality) area based on the actual data. These areas should be ignored.

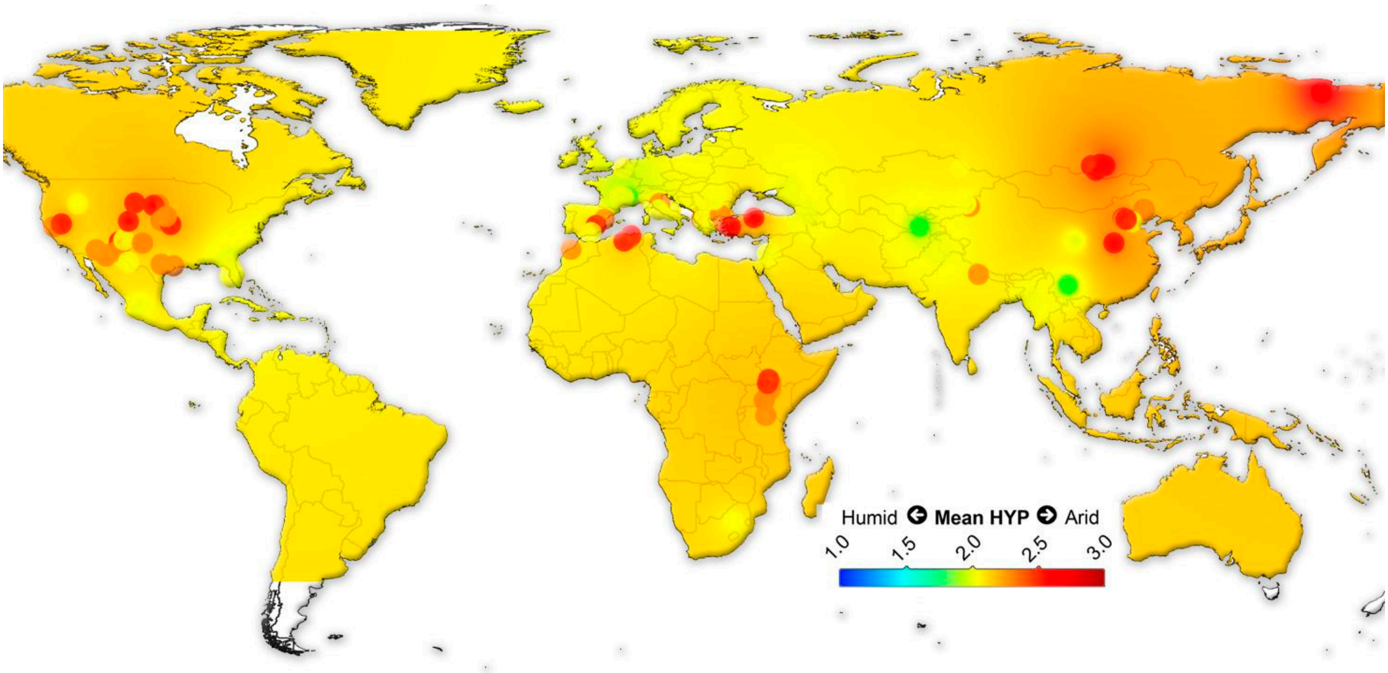

Figure S10. Spatial distribution of the large herbivorous genera mean ordinated crown height through time ranges 2.58 to 1.5 Ma in North, Central and South America, Eurasia and Africa. The mean ordinated hypsodonty map represents the paleoclimatological conditions grading from most humid (blue) to most arid (red), without the coded number given in Table S4.The mean ordinated hypsodonty values are represented by the color-coded circles indicate the spatial position of the localities that mean hypsodonty scores calculated (Table S5). IDW interpolation algorithm hypothetically interpolate no data (no locality) area based on the actual data. These areas should be ignored.

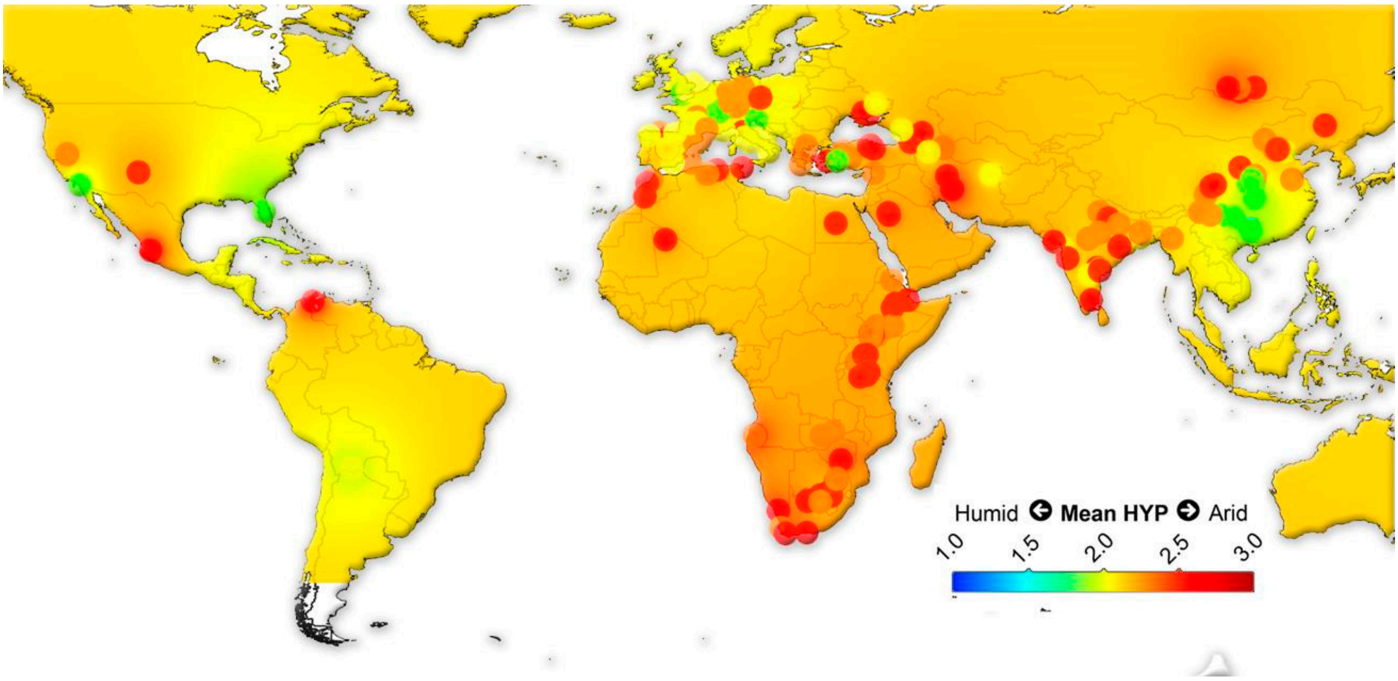

Figure S11. Spatial distribution of the large herbivorous genera mean ordinated crown height through time ranges 1.5 Ma to recent in North, Central and South America, Eurasia and Africa. The mean ordinated hypsodonty map represents the paleoclimatological conditions grading from most humid (blue) to most arid (red), without the coded number given in Table S4. The mean ordinated hypsodonty values are represented by the color-coded circles indicate the spatial position of the localities that mean hypsodonty scores calculated (Table S5). IDW interpolation algorithm hypothetically interpolate no data (no locality) area based on the actual data. These areas should be ignored.

References

Alberdi, M. T, Arroyo-Cabrales, J., Marín-Leyva, A. H., and Polaco, O. J. (2014). Study of Cedral horses and their place in the Mexican Quaternary. Rev Mex de Cienc Geol 31:221–237.

Arambourg, C. Les Vertébrés du Pléistocène de l'Afrique du Nord. *Archives du Muséum national d'Histoire Naturelle* **1970**, *10*, 1–127.

Azzaroli, A., 1990. The genus Equus in Europe. In: European Neogene Mammal Chronology. Lindsay, E.H., Fahlbusch, V., Mein, P. Eds.; Plenum Press: New York, NY, USA, 1990; 339-355.

Azzaroli, A. (1995). A synopsis of the Quaternary species of *Equus* in North America. Boll Soc Paleontol Ital. 34:205–221.

Azzaroli, A. (1998). The genus *Equus* in North America – The Pleistocene species. Paleontographia Italica 85:1–60.

Azzaroli, A., and Voorhies, M. (1993). The genus *Equus* in North America: The Blancan species. Palaeontographia Italica. 80, 175–198.

Bagtache, B.; Hadjouis, D.; Eisenmann, V. Presence d'un *Equus* caballine (*E. algericus* n. sp.) et d'une autre espece nouvelle d'*Equus* (*E. melkiensis* n. sp.) dans l'Atérien des Allbroges, Algerie. *Comptes Rendus des Séances de l'Académie des Sciences, Paris, Série II* **1984**, *298*, 609–612.

Barnosky, C. Response Of Vegetation To Climatic Changes Of Different Duration In The Late Neogene. *Trends in Ecology & Evolution* 1987, *2*, 247–250.

Barrón-Ortiz, C. I., Rodrigues, A. T., Theodor, J. M., Kooyman, B. P., Yang, D. Y., and Speller, C. F. (2017). Cheek tooth morphology and ancient mitochondrial DNA of late Pleistocene horses from the western interior of North America: implications for the taxonomy of North American Late Pleistocene *Equus*. PLoS ONE 12(8): e0183045. https://doi.org/10.1371/journal.pone.0183045

Barrón-Ortiz, C. I., Avilla, L. S., Jass, C. N., Bravo-Cuevas, V. M., Machado, H., and Mothé, D. (2019). What is *Equus*? Reconciling taxonomy and phylogenetic analyses. Frontiers in Ecology and Evolution. 7, Article 343. https://doi.org/10.3389/fevo.2019.00343.

Bennett, D. K. (1980). Stripes do not a zebra make. Part I: A cladistic analysis of *Equus*. Syst. Zool. 29, 272–287.

Bernor R.L., Heissig K. & Tobien H. (1987) - Early Pliocene Perissodactyla from Sahabi, Libya. In: Boaz N.T., de Heinzelin J., Gaziry W. & El-Arnauti A. (Eds) – Neogene Geology and Paleontology of Sahabi: 233-254. Alan Liss, New York.

Bernor, R.L.; Kaiser, T. Systematics and Paleoecology of the Earliest Pliocene Equid, *Eurygnathohippus hooijeri* n. sp. from Langebaanweg, South Africa. *Mitteilungen aus dem Hamburischen Zoologischen Museum und Institut*, **2006**, *103*, 147-183.

Bernor, R.L.; Armour-Chelu M.; Gilbert H.; Kaiser T.; Schulz. E. Equidae. In *Cenozoic Mammals of Africa*; Werdelin L., Sanders W. Eds.; University of California Press, Berkeley, California, USA, 2010; pp 685-721.

Bernor, R.L.; Sen, S. The Early Pliocene *Plesiohipparion* and *Proboscidipparion* (Equidae, Hipparionini) from Çalta, Turkey (Ruscinian Age, c. 4.0 Ma). *Geodiversitas* **2017**, *39*, 285–314.
Bernor, R.L.; Cirilli, O.; Jukar, A.M.; Potts, R.; Buskianidze, M.; Rook, L. Evolution of Early *Equus* in Italy, Georgia, the Indian Subcontinent, East Africa, and the Origins of African Zebras. *Frontiers in Ecology and Evolution* **2019**, *7*, 166.
Bernor, R.L.; Kaya, F.; Kaakinen, A.; Saarinen, J.; Fortelius, M. Old World hipparion evolution, biogeography, climatology and ecology. *Earth Science Reviews* **2021**, *221*, 103748.
Bertini, A. Pliocene To Pleistocene Palynoflora And Vegetation In Italy: State Of The Art. Quaternary International 2010, 225, 5–24.
Boulbes, N.; Van Asperen, E.N. Biostratigraphy and palaeoecology of European *Equus*. *Frontiers in Ecology and Evolution* **2019**, *7*, 301.
Brown, K. E., Akersten, W. A., and Scott, E. (2015). *Equus occidentalis* Leidy from "Asphalto," Kern County, California. Science Series. Natural History Museum of Los Angeles County 42, 81–89.
Churcher, C.S.; Richardson. M.L. Equidae. In Evolution of African Mammals; Maglio V.J., Cooke H.S.B., Eds.; Harvard University Press: Cambridge, USA, 1978; pp 379–422.
Churcher, C. S., and D. A. Hooijer. 1980. The Olduvai zebra *(Equus oldowayensis)* from the later Omo beds, Ethiopia. *Zoologische Mededelingen* 55:265–280.
Cirilli O.; Zouhri, S.; Boughabi, S.; Benvenuti, M.; Bernor, R.L.; Papini, M.; Rook, L. The hipparionine horses (Perissodactyla: Mammalia) from the Late Miocene of Tizi N'Tadderht (southern Ouarzazate basin; Central High Atlas; Morocco). *Riv. It. Paleontol. Strat.* **2020**, *126*, 1–12.
Cirilli, O., Pandolfi, L., Rook, L., and Bernor, R. L. (2021a). Evolution of Old World *Equus* and Origin of the Zebra-Ass Clade. *Sci. Rep.* 11, 1–11, doi:10.1038/s41598-021-89440-9.
Cirilli, O.; Saarinen, J.; Pandolfi, L.; Rook, L.; Bernor R.L. (2021b) An updated review on *Equus stenonis* (Mammalia, Equidae): new implications for the European Early Pleistocene *Equus* taxonomy and paleoecology, and remarks on the Old World *Equus* evolution. *Quat. Sci. Rev. 269*, 107155.
Combourieu–Nebout, N.; Bertini, A.; Russo–Ermolli, E.; Peyron, O.; Klotz, S.; Montade, V.; Fauquette, S.; Allen, J.; Fusco, F.; Goring, S. et al. Climate Changes In The Central Mediterranean And Italian Vegetation Dynamics Since The Pliocene. Review of Palaeobotany and Palynology 2015, 218, 127–147
Cooke, H.B.S. 1963. Pleistocene mammal faunas of Africa, with particular reference to southern Africa; pp. 65–116 in F. C. Howell and F. Bourlière (eds.), *African Ecology and Human Evolution.* Viking Fund Publications in Anthropology, New York.
Cope, E.D. A contribution to the vertebrate paleontology of Texas. *Proc. Amer. Philos. Soc.* **1892**, *30*, 123-131.
Dalquest, W. W. (1978). Phylogeny of American horses of Blancan and Pleistocene age. Acta Zool. Fennica. 15, 191–199.
Dalquest, W. W., and Schultz, G. E. (1992). Ice Age Mammals of Northwestern Texas. Wichita Falls, TX: Midwestern State University Press.
Downs, T., and Miller, G. J. (1994). Late Cenozoic equids from the Anza-Borrego Desert of California. Nat. Hist. Mus. Los Angeles Co., Contrib. Sci. 440: 1–90.
Eisenmann, V. Le protostylide: Valeur systematique et signification phylétique chez les espèces actuelles et fossiles du genre *Equus* (Perissodactyla, Mammalia). *Zeitschrift für Säugetierkunde* **1976**, 41, 349–365.
Eisenmann, V. *Equus capensis* (Mammalia, Perissodactyla) from Elandsfontein. *Palaeontologia Africana* **2000**, *36*, 91–96.
Eisenmann, V.; Helmer, D.; Segui, M.S. The big *Equus* from Geometric Kebaran of Umm el Tlel, Syria: *Equus valeriani*, *Equus capensis* or *Equus caballus*? Proocending of the fifth international symposium on the archaeozoology of southwestern Asia and adjacent areas 2002, 62-73.
Eisenmann, V.; Kuznetsova, T.V. Early Pleistocene equids (Mammalia, Perissodactyla) of Nalaikha (Mongolia) and the emergence of modern *Equus* Linnaeus, 1758. *Geodiversitas* **2004**, *26*, 535–561.
Eisenmann, V.; Geraads, D. *Hipparion pomeli* sp. nov. from the late Pliocene of Ahl al Oughlam, Morocco, and a revision of the relationships of Pliocene and Pleistocene African hipparions. *Palaeo. Afr*. **2006**, *42*, 51–98.
Eisenmann, V., Howe, J., Pichardo, M. (2008). Old World hemiones and New World slender species (Mammalia, Equidae). Palaeovertebrata 36:159–233. https://doi.org/10.18563/pv.36.1-4.159-233
Eisenmann, V. (2010). *Sussemionus*, a new subgenus of *Equus* (Perissodactyla, Mammalia). Comptes Rendus Biologies 333:235–240. https://doi.org/10.1016/j.crvi.2009.12.013
Eisenmann, V.; Vasiliev, S. Unexpected finding of a new *Equus* species (Mammalia, Perissodactyla) belonging to a supposedly extinct subgenus in Late Pleistocene deposits of Khakassia (southwestern Siberia). *Geodiversitas*, **2011**, *33*, 519–530
Eronen, J.; Fortelius, M.; Micheels, A.; Portmann, F.; Puolamaki, K.; Janis, C. Neogene Aridification Of The Northern Hemisphere. *Geology* 2012, *40*, 823–826.
Fedorov, A.; Brierley, C.; Lawrence, K.; Liu, Z.; Dekens, P.; Ravelo, A. Patterns And Mechanisms Of Early Pliocene Warmth. Nature 2013, 496, 43–49.
Fortelius, M., and Solounias, N. (2000). Functional characterization of ungulate molars using the abrasion-attrition wear gradient: a new method for reconstructing paleodiets Am. Museum Novit. 3301, 1–36.
Gervais, P. Recherches sur les Mammiferes fossiles de l'Amerique meridionale. P. Bertrand, Librarie-Editeur: Paris, France, **1855**. pp 1-63.
Gidley, J. W. (1900). A new species of Pleistocene horse from the Staked Plains of Texas. Bull Am Mus Nat 13, 111–116.
Gidley, J. W. (1901). Tooth characters and revision of the North American species of the genus *Equus*. Bull Am Mus Nat 14, 91–142.
Gkeme, A.G.; Koufos, G.D.; Kostopoulos, D.S. Reconsidering the Equids from the Early Pleistocene Fauna of Apollonia 1 (Mygdonia Basin, Greece). *Quaternary* **2021**, *4*, 12.
Gromova, V.I**.** O novoj iskopaemoj loshadi iz Srednej Azii. Doklady AN SSSR **1946,** vol. 54(4): 349-352.

Gromova, V.I. Istorija loshadej (roda *Equus*) v Starom Svete. Chast' 1. Obzor i opisanie form. *Trudy paleontologicheskogo Instituta* **1949**, *17*, 1–373.

Groves, C.P. 2002. Taxonomy of living Equidae. *Report* 8:94–107. Gland, Switzerland, and Cambridge, IUCN/SCC Equid Specialist Group, IUCN.

Groves, C. P., and O. A. Ryder. 2000. Systematics and phylogeny of the horse; pp. 1–24 in A. T. Bowling and A. Ruvinsky (eds.), *The Genetics of the Horse*. CABI Publishing, Oxford.

Harington, C. R. (1990). Vertebrates of the Last Interglaciation in Canada: A review, with new data. Géogr Phys Quatern 44(3), 375–387. https://doi.org/10.7202/032837ar

Harington, C. R. (2011). Pleistocene vertebrates of the Yukon Territory. Quat Sci Rev 30, 2341–2354. https://doi.org/10.1016/j.quascirev.2011.05.020

Hay, O. P. (1915). Contributions to the knowledge of the mammals of the Pleistocene of North America. Proc US Natl Mus 48, 515–575. https://doi.org/10.5479/si.00963801.48-2086.515

Hay, O. P. (1917). Description of a new species of extinct horse, *Equus lambei*, from the Pleistocene of Yukon Territory. Proc US Natl Mus 53, 435-443.

Head, M.; Gibbard, P. Early–Middle Pleistocene Transitions: Linking Terrestrial And Marine Realms. Quaternary International 2015, 389, 7–46.

Heintzman, P. D., Zazula, G. D., MacPhee, R. D. E., Scott, E., Cahill, J. A., McHorse, B. K., Kapp, J. D., Stiller, M., Wooller, M. J., Orlando, L., Southon, J., Froese, D. G., Shapiro, B. (2017). A new genus of horse from Pleistocene North America. eLife 6:e29944. https://doi.org/10.7554/eLife.29944

Hibbard, C. W. (1955). Pleistocene vertebrates from the Upper Becerra Formation, Valley of Tequixquiac, Mexico, with notes on other Pleistocene forms. Contrib Mus Paleont, Univ Michigan. 12, 47–96.

Hoffstetter, R. (1950). Algunas observaciones sobre los caballos fósiles de America del Sur. Amerhippus gen. nov. Bol. Inform. Cient. Nacional. 3, 426–454.

Hooijer, D. A. 1976. The late Pliocene Equidae of Langebaanweg, Cape Province, South Africa. *Zoologische Verhandelingen* 148:3–39.

Hopwood, A.T. Die fossilen Pferde von Oldoway. *Wissenschaften Ergebnisse Oldoway Expedition* **1937**, *4*, 112–136.

Hulbert, R.C., 1989. Phylogenetic interrelationships and evolution of North American late Neogene Equidae. In: Prothero, D.R., Schoch, R.M. (Eds.), The Evolution of Perissodactyls. Clarendon Press, Oxford, pp. 176–196.

Hulbert, R.C., 1993. Taxonomic evolution in North American Neogene horses (Subfamily Equinae): the rise and fall of an adaptive radiation. Paleobiology 19, 216–234.

Hulbert, R. C., Jr. (1995). *Equus* from Leisey Shell Pit 1A and other Irvingtonian localities from Florida. Bull. Florida Mus. Nat. Hist. 37 Pt. II (17), 553–602.

Hulbert, R.C., MacFadden, B.J., 1991. Morphological transformation and cladogenesis at the base of the adaptive radiation of Miocene hypsodont horses. Am. Mus. Novit. 3000, 1–61.

Hulbert, R.C.; Harrington, R. An early Pliocene hipparionine horse from the Canadian Arctic. *Paleontology* **1999**, *42*, 1017–1025

Kaiser, T. M., and T. A. Franz-Odendaal. 2004. A mixed feeding *Equus* species from the Middle Pleistocene of South Africa. *Quaternary Research* 62:316–323

Koufos G.D.; Vlachou, T. 2005. Equidae (Mammalia, Perissodactyla) from the late Miocene of Akkasdagi, Turkey. In: Sen, S. (Eds.), Geology, Mammals and Environments at Akkasdagi, Late Miocene of Central Anatolia. Geodiversitas 27, pp. 633–705.

Koufos G.D.; Vlachou, T.; Gkeme, A. The Fossil Record of Equids (Mammalia: Perissodactyla: Equidae) in Greece. In: *Fossil Vertebrates of Greece Vol. 2*; Vlachos, E., Eds.; Springer, Dortrecht, The Netherlands, 2021; pp 351-401.

Kurtén, B., and Anderson, E. (1980). Pleistocene Mammals of North America. New York City, USA: Columbia University Press.

Kuzmina I.E. Nekotorye dannye o mlekopitajushchikh Severnogo Urala v pozdnem Plejstotsene. *Bjulljuten kommissii po izucheniju chetvertichnogo perioda* **1975**, vol. 43: 63-77.

Janis, C.M., 1984. The significance of fossil ungulate communities as indicators of vegetation structure and climate. In: Brenchley, P.J. (Ed.), Fossils and Climate. John Wiley and Sons, New York, pp. 85–104.

Janis, C.M., 1993. Tertiary mammal evolution in the context of changing climates, vegetation, and tectonic events. Annu. Rev. Ecol. Syst. 24, 467–500.

Jukar, A.M., Sun, B., Nanda, A.C., Bernor, R.L. The first occurrence of *Eurygnathohippus* (Mammalia, Perissodatyla, Equidae) outside Africa and its biogeographic significance. *Boll. Soc. Paleo. Ital.* **2019**, *58*, 171–179.

Lance, J.F. Palaeontología y estratigrafía del Pliocene de Yepómera, estado de Chihuahua 1a Parte: Equidos, excepto Neohipparion. *Instituto de Geología, Universidad Nacional Autónoma de México* **1950**, *54*, 1–81

Lazarev, P.A. Antropogenovye Loshadi Iakutii. Nauka Moscow 1980

Lazarev P.A. Large mammals of Anthropogene in Yakutia (in Russian). Nauka, Novosibirsk, **2008**, pp.160.

Lund, P.W. Meddelelse af det ubbytte de i 1844 undersögte Knoglehuler have avgivet til Kundskaben om Brasiliens Dyreverden för sidste Jordomvaeltning. *Ebenda* **1846**, *12*, 1–94

Lundelius, E. L., Jr. (1972b). Fossil vertebrates from the late Pleistocene Ingleside fauna, San Patricio County, Texas. Bureau of Economic Geology, The University of Texas at Austin, Report of Investigations No. 77, 1–74.

Machado, H.; Grillo, O.; Scott, E.; Avilla, L. Following the footsteps of the South American *Equus*: are autopodia taxonomically informative? *J. Mammal. Evol.* **2017**, *25*, 397–405.

Machado, H.; Avilla, L. The Diversity of South American *Equus*. Did Size Really Matter? *Frontiers in Ecology and Evolution* **2019**, *7*, 235.

MacFadden, B.J. Systematics and phylogeny of *Hipparion*, *Neohipparion*, *Nannippus*, and *Cormohipparion* (Mammalia, Equidae), from the Miocene and Pliocene of the New World. *Bulletin of the American Museum of Natural History* **1984**, *179*, 196.

MacFadden, B.J., 1992. Fossil Horses: Systematics, Paleobiology, and Evolution of the Family Equidae. Cambridge University Press, Cambridge

Marín-Leyva1, A.H.; Arroyo-Cabrales, J.; García-Zepeda, M.L.; Ponce-Saavedra, J.; Schaaf, P.; Pérez-Crespo, V.P.; Pedro Morales-Puente, P.; Cienfuegos-Alvarado, E.; Alberdi, M.T.; Feeding ecology and habitat of Late Pleistocene Equus horses fromwest-central Mexico using carbon and oxygen isotopes variation. *Revista Mexicana de Ciencias Geologicas* **2019**, *33*, 157-169.

Michaux, J.; Suc, J.; Vernet, J. Climatic Inference From The History Of The Taxodiaceae During The Pliocene And The Early Pleistocene In Western Europe. Review of Palaeobotany and Palynology 1979, 27, 185–191.

Mihlbachler, M.C., Rivals, F., Solounias, N., Semprebon, G.M., 2011. Dietary change and evolution of horses in North America. Science 331, 1178–1181.

Mix, A. Environmental Processes Of The Ice Age: Land, Oceans, Glaciers (EPILOG). Quaternary Science Reviews 2001, 20, 627–657.

Moodley, Y., and E. H. Harley. 2005. Population structuring in mountain zebras *(Equus zebra)*: The molecular consequences of divergent demographic histories. *Conservation Genetics* 6:953–968.

Moodley, Y., I. Baumgarten, and E. H. Harley. 2006. Horse microsatellites and their amenability to comparative equid genetics. *Animal Genetics* 37:258–261

Musil, R. Die Equiden–Reste aus dem Unterpleistozän von Untermassfeld. In *Das Pleistozän von Untermassfeld bei Meiningen (Thüringen). Teil 2*; Kahlke, R.D. Eds.; Monographien, Römisch–Germanisches Zentralmuseum, Forschungsinstitut für Vor und Frühgeschichte, Liebniz, Germany, 2001; pp 557–587

Orlando, L., Ginolhac, A., Zhang, G., Froese, D., Albrechtsen, A., Stiller, M., et al. (2013). Recalibrating *Equus* evolution using the genome sequence of an early Middle Pleistocene horse. Nature 499, 74–78.

Osborn, H.F., 1910. The Age of Mammals. MacMillan, New York.

Owen, R. (1869). On fossil remains of Equines from Central and South America referable to *Equus conversidens* Ow., *E. tau* Ow., and *E. arcidens* Ow. Philos Trans R Soc Lond. 159, 559–573.

Pichardo, M. (2000). Valsequillo biostratigraphy III: Equid ecospecies in paleoindian sites. Anthrop. Anz. 58, 275–298.

Pichardo, M. (2004). Review of horses in paleoindian sites of the Americas. Anthrop. Anz. 62, 11–35.

Pichardo, M. (2008). Identification guide for horses from North American paleoindian time. Minneapolis, MN: Copyright © Mario Pichardo (limited edition of 52 copies).

Pomel, A. Les equidés: Carte géologie de l'Algérie. *Monographies de Paléontologie* **1897**, *12*, 1–44

Prado, J.L.; Alberdi, M.T. *Fossil Horses of South America*. Springer International Publishing, (2017), pp 1-150.

Priego-Vargas, J., Bravo-Cuevas, V. M., and Jiménez-Hidalgo, E. (2017). Revisión taxonómica de los équidos del Pleistoceno de México con base en la morfología dental. Rev Bras Paleontol 20, 239–268. https://doi.org10.4072/rbp.2017.2.07

Qiu, Z. ; Huang, W.; Guo, Z. The Chinese hipparionine fossils. *Palaeontologia Sinica New Series C*, **1987**, *175*, 1-250.

Quinn, J. H. (1957). Pleistocene Equidae of Texas. Bureau of Economic Geology, University of Texas. 33, 5–51.

Radinsky, L., 1983. Allometry and reorganization of horse skull proportions. Science 221, 1189–1191.

Radinsky, L., 1984. Ontogeny and phylogeny in horse skull evolution. Int. J. Org. Evol. 38, 1–15.

Savage, D. E. (1951). Late Cenozoic vertebrates, San Francisco Bay Region. Bull Dep Geo Scien. 28, 215–314.

Schultz, J. R. (1936). *Plesippus francescana* (Frick) from the late Pliocene, Coso Mountains, California, with a review of the genus *Plesippus*. Contributions to Palaeontology. Publications of the Carnegie Institution of Washington 1 (473), 1–13.

Scott, W.B., 1937. A History of Land Mammals in the Western Hemisphere. The MacMillian Company, New York.

Scott, E. (2004). Chapter 20. Pliocene and Pleistocene horses from Porcupine Cave. In: Barnosky, A. (Ed.), Biodiversity Response to Climate Change in the Middle Pleistocene: The Porcupine Cave Fauna from Colorado. University of California Press, Berkeley. pp. 264–279.

Scott, E. (2005). *Equus idahoensis* from the Pliocene of Arizona, and its role in plesippine evolution in the American southwest. Available at http://www.sbcounty.gov/museum/discover/divisions/geo/pdf/SVP_2005b_EquusIdahoensis.pdf.

Scott, E., Stafford, T. W. Jr, Graham, R .W., and Martin, L. D. (2010). Morphology and metrics, isotopes and dates: determining the validity of *Equus laurentius* Hay, 1913. J Vertebr Paleontol. 30, 1840–1847. doi:10.1080/02724634.2010.520780.

Semprebon, G.M., 2002. Advances in the Reconstruction of Extant Ungulate Ecomorphology with Applications to Fossil Ungulates (Ph.D. Dissertation) University of Massachusetts, Amherst.

Semprebon, G.M., Godfrey, L.R., Solounias, N., Sutherland, M.R., Jungers, W.L., 2004. Can low-magnification stereomicroscopy reveal diet? J. Hum. Evol. 47, 115–144. Semprebon, G.M., Rivals, F., 2007. Was grass more prevalent in the pronghorn past? An assessment of the dietary adaptations of Miocene to recent Antilocapridae (Mammalia: Artiodactyla). Palaeogeogr. Palaeoclimatol. Palaeoecol. 253, 332–347.

Semprebon, G.M., Rivals, F., and Janis, C.M., 2019. The role of grass vs. exogenous abrasives in the paleodietary patterns of North American ungulates. Front. Ecol. Evol. https://doi.org/10.3389/fevo.2019.00065

Sher A.V. *Mlekopitaiushchie i Stratigrafia Pleistotsena Krainego Severo-Vostoka SSSR i Severnoi Ameriki*. Nauka, Moskva, Russia, 1971.

Simpson, G.G., 1944. Tempo and Mode in Evolution. Columbia University Press, New York.

Skinner, M. F. (1972). Order Perissodactyla. In: Skinner, M. F., and Hibbard, C. W. (Eds.) Early Pleistocene Pre-Glacial and Glacial Rocks and Faunas of North-Central Nebraska. Bull. Amer. Mus. Nat. Hist. 148, 1–148.

Solounias, N., Semprebon, G., 2002. Advances in the reconstruction of ungulate ecomorphology with application to early fossil equids. Am. Mus. Novit. 3366, 1–49.

Stirton, R.A., 1947. Observation of evolutionary rates in hypsodonty. Evolution 1, 32–41.

Suc, J. Origin And Evolution Of The Mediterranean Vegetation And Climate In Europe. Nature 1984, 307, 429–432.

Suc, J.; Popescu, S. Pollen Records And Climatic Cycles In The North Mediterranean Region Since 2.7 Ma. Geological Society, London, Special Publications 2005, 247, 147–158.

Sun, B.; Deng, T. The *Equus* Datum and the Early Radiation of Equus in China. *Frontiers in Ecology and Evolution* **2019**, *7*, 429.

Thomas, P. Recherches stratigraphiques et paleontologiques sur quelques formations d'eau douce de l'Algerie. *Memoire de la Societe geologique de France* **1884**, *3*, 1-50.

van Asperen, E. N., Stefaniak, K., Proskurnyak, L., and Ridush, B. (2012). Equids from Emine-Bair-Khosar cave (Crimea, Ukraine): co-occurrence of the stenonid Equus hydruntinus and the caballoid E. ferus latipes based on skull and postcranial remains. Palaeontol. Electron. 15:28. doi: 10.26879/280

van der Made, J.; Boulaghraif, K.; Chelli Cheheb, R.; Caceres, I.; Harichane, Z.; Sahnouni, M.. The last North African hipparions – hipparion decline and extinction follows a common pattern. *N. Jb. Geol. Palaont. Abh.* **2022**, *303*, 39-97

Vershinina, A. O., Heintzman, P. D., Froese, D. G., Zazula, G. D., Cassatt-Johnstone, M., Dalén, L., Der Sarkissian, C., Dunn, S. G., Ermini, L., Gamba, C., Groves, P., Kapp, J. D., Mann, D. H., Seguin-Orlando, A., Southon, J., Stiller, M., Wooller, M. J., Baryshnikov, G., Gimranov, D., Scott, E., Hall, E., Hewitson, S., Kirillova, I., Kosintsev, P., Shidlovsky, F., Tong, H.-W., Tiunov, M. P., Vartanyan, S., Orlando, L., Corbett-Detig, R., MacPhee, R. D., and Shapiro, B. (2021). Ancient horse genomes reveal timing and extent of dispersals across the Bering Land Bridge. Mol Ecol 30(23), 6144–6161. https://doi.org/10.1111/mec.15977

Webb, S.D., 1977. A history of savanna vertebrates in the New World. Part I: North America. Annu. Rev. Ecol. Syst. 8, 355–380.

Webb, S.D., 1983. The rise and fall of the late Miocene ungulate fauna in North America. In: Nitecki, M.H. (Ed.), Coevolution. University of Chicago Press, Chicago, pp. 267–306.

Webb, S.D., Opdyke, N.D., 1995. Global climatic influence on Cenozoic land mammal faunas. National Research Council Panel on Effects of Past Global Change on Life (Eds.) Effects of past global change on life. National Academies Press, Washington, pp. 184–208.

Weinstock, J., Willerslev, E., Sher, A., Tong, W., Ho, S. Y. W., Rubenstein, D., Storer, J., Burns, J., Martin, J., Bravi, C., Prieto, A., Froese, D., Scott, E., Xulong, L., Cooper, A. (2005). Evolution, systematics, and phylogeography of Pleistocene horses in the new world: a molecular perspective. PLoS Biol 3(8), 1373–1379. https://doi.org/10.1371/journal.pbio.0030241

Winans, M. C. (1985). Revision of North American fossil species of the genus *Equus* (Mammalia: Perissodactyla: Equidae). Ph.D. Dissertation, University of Texas at Austin.

Winans, M. C. (1989). A quantitative study of the North American fossil species of the genus *Equus*. In: Prothero, D. R., and Schoch, R. M. (Eds.), The evolution of Perissodactyls. New York: Clarendon Press. pp. 262–297.

Yuan, J.; Sheng G.; Preick M.; Sun B.; Hou X.; Chen S.; Taron, U.H.; Barlow, A.; Wang, L.; Hu, J.; Deng, T.; Lai, X.; Hofreiter, M. Mitochondrial genomes of Late Pleistocene caballines horses from China belong to a separate clade. *Quat. Sci. Rev.* **2020**, *250*, 106691

Zouhri, S., Bensalmia, A., 2005. R´evision Syst´ematique des Hipparion sensu lato (Perissodactyla, Equidae) de l'Ancient Monde. Estud. Geo. 61, 61–99.
